# An Unusual Diterpene—Enhygromic Acid and Deoxyenhygrolides from a Marine Myxobacterium, *Enhygromyxa* sp.

**DOI:** 10.3390/md15040109

**Published:** 2017-04-06

**Authors:** Tomohiko Tomura, Shiori Nagashima, Satoshi Yamazaki, Takashi Iizuka, Ryosuke Fudou, Makoto Ojika

**Affiliations:** 1 Graduate School of Bioagricultural Sciences, Nagoya University, Chikusa-ku, Nagoya 464-8601, Japan; ttomura@agr.nagoya-u.ac.jp (T.T.); nagashima.shiori@d.mbox.nagoya-u.ac.jp (S.N.); yamazaki.satoshi@a.mbox.nagoya-u.ac.jp (S.Y.); 2 Institute for Innovation, Ajinomoto Co., Inc., Kawasaki, Kanagawa 210-8681, Japan; takashi_iizuka@ajinomoto.com; 3 R & D Planning Department, Ajinomoto Co., Inc., Chuo-ku, Tokyo 104-8315, Japan; ryosuke_fudou@ajinomoto.com

**Keywords:** marine myxobacterium, diterpene, NGF-enhancing activity, cytotoxicity, antibacterial activity

## Abstract

Three new compounds, enhygromic acid (**1**) and deoxyenhygrolides A (**2**) and B (**3**), were isolated from a marine myxobacterium, *Enhygromyxa* sp. Compound **1** was found to be an acrylic acid derivative with a rare polycyclic carbon skeleton, decahydroacenaphthylene, by spectroscopic analyses. Compounds **2** and **3** were deoxy analogs of the known γ-alkylidenebutenolides, enhygrolides. Compound **1** exhibited cytotoxicity against B16 melanoma cells and anti-bacterial activity against *Bacillus subtilis*, and enhanced the NGF-induced neurite outgrowth of PC12 cells.

## 1. Introduction

**Figure 1:**
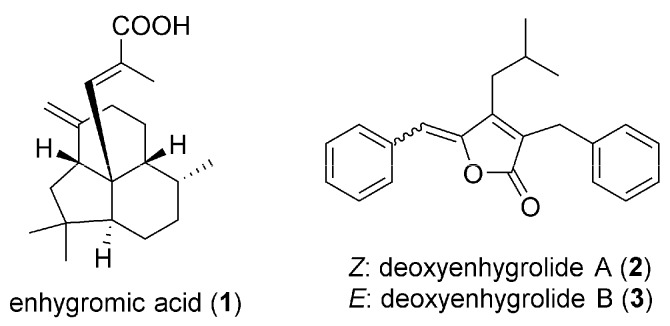
Structures of **1**–**3**.

Myxobacteria are unique δ-proteobacteria that glide and aggregate to form swarms and multicellular fruiting bodies [1]. Myxobacteria were recently recognized as potential sources of novel secondary metabolites such as polyketides and non-ribosomal peptides as well as their hybrid compounds [2,3]. Marine-derived myxobacteria are especially attractive [4] because their polyketide synthase gene sequences are unique [5], although they are more difficult to cultivate compared to their terrestrial counterparts. In recent years, some bioactive compounds were discovered from marine myxobacteria including haliangicins [6,7] and haliamide [8] from *Haliangium ochraceum*; miuraenamides produced by *Paraliomyxa miuraensis* [9,10]; and salimabromides [11], salimyxins [12], and enhygrolides [12] produced by *Enhygromyxa salina* [13]. Our group has been searching for new myxobacteria species in marine environment for decades [14]. Our research on marine myxobacterial secondary metabolites led to the discovery of three new compounds, enhygromic acid (**1**) and deoxyenhygrolides A (**2**) and B (**3**) (Figure 1), from a new species within the genus *Enhygromyxa*. Here, we report the isolation, structural elucidation, and biological activities of these compounds, one of which is an acrylic acid derivative with a rare tricyclic carbon skeleton.

## 2. Results and Discussion

### 2.1. Isolation and Structural Elucidation of ***1**–**3***

**Figure 2:**
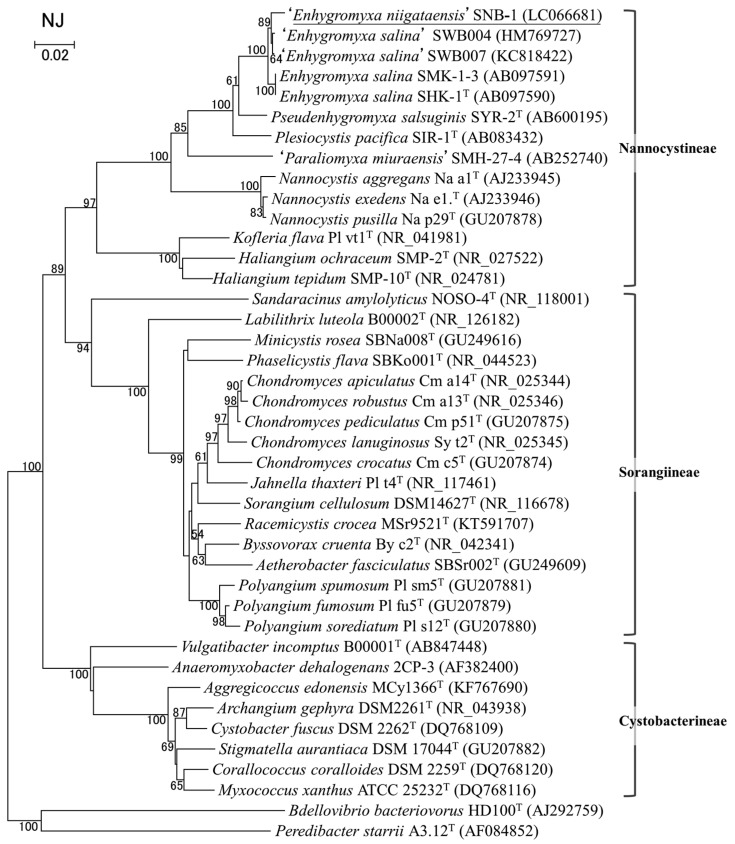
Phylogenetic tree of myxobacteria. Neighbor-joining (NJ) tree based on 16S rRNA gene sequences shows the positions of *Enhygromyxa* sp. SNB-1 (tentative name: *E. niigataensis*, top) in the order Myxococcales. The bar represents 20 nt substitutions per 1000 sites. Bootstrap values (>50%) based on 1000 replications are shown at branch nodes. Three suborders in the order Myxococcales are also indicated. The two species at the bottom were used as outgroups.

*Enhygromyxa* sp. SNB-1 was found to be a new species based on the 16S rRNA gene sequence (97% similarity to the closest type strain) and tentatively designated as *Enhygromyxa niigataensis* (Figure 2).

The strain was cultured for three weeks in VY/4-SWS (2% NaCl) liquid medium supplemented with 100 mM sodium acetate. Twenty-one liters of cultures containing bacterial cells were extracted with acetone, and aqueous acetone extracts were subsequently extracted with EtOAc. The resulting extract was separated by flash column chromatography on silica gel (hexane–EtOAc), and each fraction was subjected to an MTT assay to determine cytotoxicity against B16 melanoma cells. After a two-step purification of the active fractions by HPLC, Compound **1** (6.9 mg) was obtained as a cytotoxic substance. Meanwhile, Compounds **2** (4.5 mg) and **3** (17.6 mg) were obtained from another fraction.

**Figure 3:**
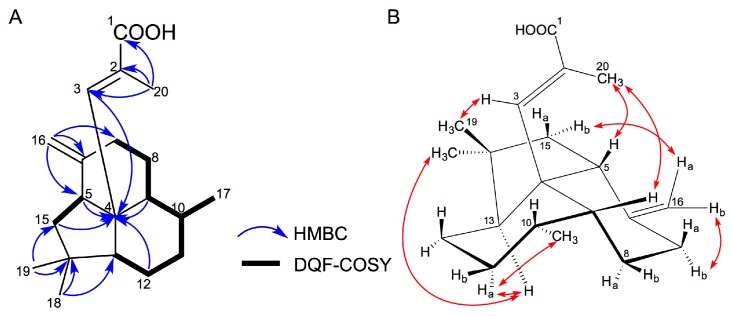
Two-dimensional NMR correlations for **1**. (**A**) DQF-COSY (bold bonds) and HMBC correlations (arrows). (**B**) Key NOESY correlations (double-headed red arrows).

The molecular formula of enhygromic acid (**1**) was determined to be C_20_H_30_O_2_ by high-resolution electrospray ionization mass spectrometry (ESIMS) analysis using the molecular ion at *m/z* 303.2299 [M + H]^+^ (calculated for C_20_H_31_O_2_: 303.2319). The infrared (IR) spectrum showed a strong absorption at 1685 cm^−1^ and a very broad band between 3640 and 2390 cm^−1^, and the ^13^C nuclear magnetic resonance (NMR) spectrum displayed the carbonyl carbon signal at *δ*_C_ 170.2, suggesting the presence of a carboxy group. The ^1^H and ^13^C NMR data are listed in Table 1. The two-dimensional NMR correlations of Compound **1** are summarized in Figure 3. Two partial structures, –CH_2_–CH< and –CH_2_–CH_2_–CH–CH(CH_3_)–CH_2_–CH_2_–CH<, were revealed by DQF-COSY correlations. The connectivity of these substructures and the five quaternary carbons were determined by interpretation of the HMBC spectrum (Figure 3A). The elucidated planar structure of **1** contained a fused tricyclic skeleton linked with an α-methylacrylic acid chain (Figure 3). The geometry of the double bond at C-2 was determined to be *E* on the basis of the NOE correlations of H-20/H-5 and H-20/H-9, and the lack of the NOE of H-3/H-20 (Figure 3B). The relative configuration of 4S*,5S*,9R*,10R*,13S* was confirmed from the NOE correlations, displayed in Figure 3B.

**Figure 4:**
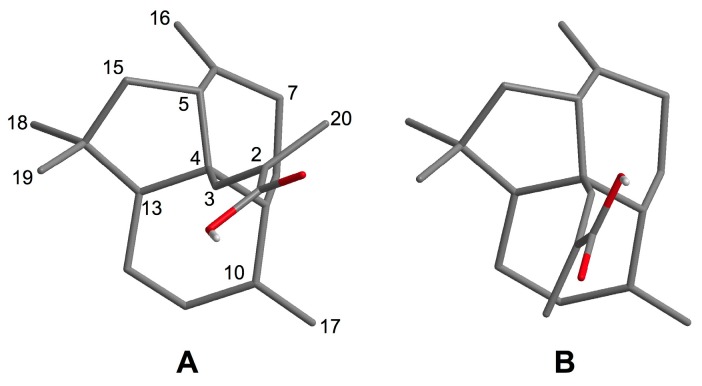
Two energy-minimized conformers **A** and **B** of **1**.

**Figure 5:**
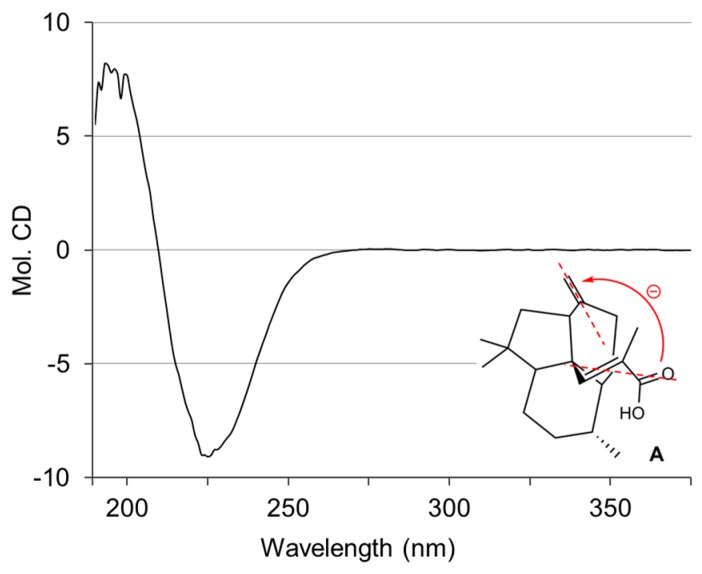
CD spectrum and the lowest energy conformer **A** of **1**.

In order to determine the absolute stereochemistry of **1**, conformational analysis was conducted by minimizing the energy of all the conformers generated by changing the torsion angle of the C-3–C-4 bond. Only two energy-minimized conformers, **A** and **B**, were obtained (Figure 4); the conformer **A** was more stable than **B** by approximately 14 kcal/mol (Figure S10). Among them, the more stable conformer **A** was strongly supported by the NOESY correlations (Figure 3B). The circular dichroism (CD) spectrum of **1** was subsequently analyzed to determine the spacial relationships between the acrylic acid moiety and the exomethylene group at C-6. On the basis of the splitted negative Cotton effect at 226 nm (Δε − 9.0) and 196 nm (Δε + 8.0) (Figure 5) and in conjunction with the above-mentioned conformational analysis, the absolute configuration of **1** was determined to be 4*S*,5*S*,9*R*,10*R*,13*S*. Interestingly, the ^13^C NMR signals of C-2 and C-3 indicated weak and broad signals, suggesting that the free rotation of the methylacrylic acid moiety extending from the center (C-4) of the fused tricyclic core is restricted by the steric repulsion between them. These findings corroborate the uniqueness of the structure of **1**.

**Figure 6:**
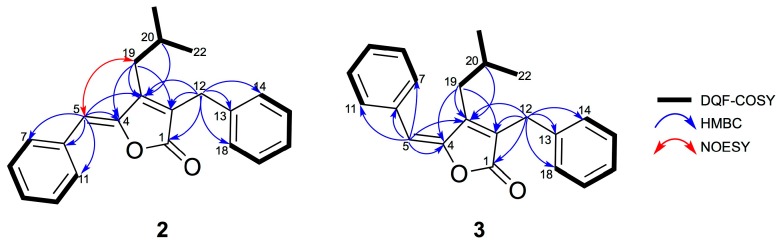
Key correlations in two-dimensional NMR spectra of **2** and **3**.

Both Compounds **2** and **3** had the molecular formula C_22_H_22_O_2_ as determined by high-resolution ESIMS. The IR absorption at 1760–1763 cm^−1^ suggests that they contain a carbonyl group. Their ^1^H and ^13^C NMR data are summarized in Table 2. Three partial structures, two C_6_H_5_– and a –CH_2_–CH(CH_3_)_2_, were obtained from DQF-COSY correlations of **2** (Figure 6). The connectivity of these substructures, five quaternary carbons (δ_C_ 127.5, 133.8, 138.4, 149.2, and 152.0), one carbonyl (δ_C_ 169.8), one methylene (δ_C_ 30.1), and one sp^2^ methine (δ_C_ 109.0), was determined on the basis of the HMBC correlations (Figure 6), furnishing the planar structure of **2**. The *Z* configuration of the C-4–C-5 double bond was determined by the NOESY correlation between H-5 and H-19. The structure of **3** was determined as an isomer of **2** in a similar manner to that for **2** (Table 2 and Figure 6). In contrast to **2**, the geometry of the double bond at C-4 was found to be *E* because of the lack of NOE correlation between H-5 and H-19, concluding that **3** is the *E* isomer of 2. Deoxyenhygrolides A (**2**) and B (**3**) are new enhygrolide analogs, where the phenolic groups in enhygrolides [12] are replaced by hydrogen atoms. Although enhygrolide A (*Z* isomer) is reported to be more stable than enhygrolide B (*E* isomer) and that their isomerization is irreversible [12], we did not observe the isomerization from **3** (*E*) to **2** (*Z*), and the yield of **3** was approximately four times that of **2**. Thus, the phenolic hydroxyl group of enhygrolides may affect the stability of the *E*/*Z* isomers for this type of compound.

### 2.2. Bioactivities of ***1**–**3***

**Figure 7:**
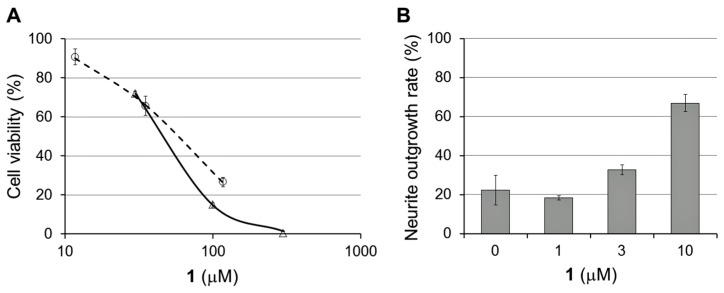
Bioactivities of enhygromic acid (**1**). (**A**) Cytotoxicity of **1** (open triangles) and paclitaxel (open circles, dotted line) against the B16 melanoma cells. (**B**) Enhancing activity of NGF-induced neurite outgrowth of PC12 cells.

Enhygromic acid (**1**) exhibited cytotoxicity against B16 melanoma cells, and its 50% inhibitory concentration (*IC*_50_) of 46 μM is comparable to that of paclitaxel (57 μM) (Figure 7A). On the other hand, against HeLa-S3 cells, **1** showed no inhibitory activity (*IC*_50_ > 30 μM) although paclitaxel was highly active (*IC*_50_ = 6.0 nM). Furthermore, **1** enhanced the nerve growth factor (NGF)-induced neurite outgrowth of PC12 cells. The neurite outgrowth (22%) induced by a trace amount (1 ng/mL) of NGF was enhanced up to 67% in a dose-dependent manner (Figure 7B). In anti-microbial tests, **1** inhibited the growth of a Gram-positive bacterium, *Bacillus subtilis*, at a minimum inhibitory concentration (*MIC*) of 8 μg/mL, but was inactive against a Gram-negative bacterium (*Escherichia coli*), fungi (*Candida rugosa*, *Aspergillus niger*, *Rhizopus oryzae*, and *Trichophyton mentagrophytes*), and an oomycete (*Phytophthora capsici*).

On the other hand, deoxyenhygrolides A (**2**) and B (**3**) did not exhibit any activities in the above-mentioned tests. They are classified into γ-alkylidenebutenolides, and some structurally related bioactive metabolites were reported, e.g., enhygrolides [12], nostoclides [15], and cyanobacterin [16]. Enhygrolide A, a phenolic congener of **2**, was reported to show an antimicrobial activity (*MIC* = 4 μg/mL) against a Gram-positive bacterium, *Arthrobacter* sp. Nostoclides are chlorinated phenols and showed a moderate cytotoxicity (*IC*_50_ = 10 μg/mL) against animal cell lines (Neuro-2a and KB cells). Cyanobacterin is a chlorinated phenyl ether and is known to inhibit the growth of cyanobacteria and green algae. The lack of the bioactivities of **2** and **3** suggests that the (halogenated) phenol group(s) may play an important role for the bioactivity of the γ-alkylidenebutenolides.

### 2.3. Putative Biosynthetic Mechanism for ***1***

**Figure 8:**
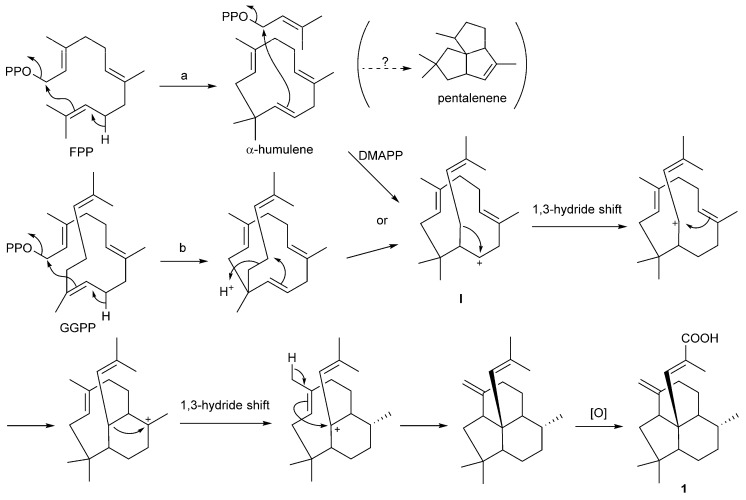
A putative biosynthetic pathway for enhygromic acid (**1**). The first step would be the cyclization of farnesyl diphosphate (FPP) to α-humulene (Route a) or geranylgeranyl diphosphate (GGPP) to a dimethylallylhumulene derivative (Route b), which are converted to the common cation intermediate **I** and led to **1** via a series of steps.

Enhygromic acid (**1**) possesses a unique fused ring structure, decahydroacenaphthylene, which is connected with α-methylacrylic acid. The carbon skeleton of **1** can be divided into four isoprene units [C-1–C-2(C-20)–C-3–C-4, C-18–C-14(C-19)–C-13–C-12, C-11–C-10(C-17)–C-9–C-8, C-7–C-6(C-16)–C-5–C-15], suggesting that this molecule is a diterpene. However, the connectivity of the first unit C-1–C-2(C-20)–C-3–C-4 is inconsistent with the head-to-tail isoprene rule. Therefore, we propose two putative pathways for the biosynthesis of **1**: (a) α-humulene formation from farnesyl diphosphate (FPP) followed by a post-prenylation; or (b) cyclization of geranylgeranyl diphosphate (GGPP) followed by the rearrangement of one isoprenyl unit (Figure 8). The resulting intermediate I undergoes repeated 1,3-hydride shift/cyclization and the oxidation of the terminal methyl group. In silico analysis was carried out on the genome sequences of *Enhygromyxa salina* DSM 15201 (= *E. salina* SHK-1**^T^** in Figure 2) [4] by antiSMASH (ver. 4.0.0) software (http://antismash.secondarymetabolites.org), revealing the presence of seven terpenoid biosynthetic gene clusters, three of which were found to contain a core gene homologous to a terpene (pentalenene) synthase gene. Since pentalenene is known to be biosynthesized from FPP via α-humulene (Figure 8), one of these genes might be responsible for the biosynthesis of **1**, and Route a is more plausible than Route b. However, unfortunately, we were unable to find any related genes such as an FPP (or GGPP) synthase gene, a prenyltransferase gene (Route a), and oxidase gene(s) inside or around these gene clusters to support the biosynthetic route. The biosynthetic mechanism for the construction of this unique carbon skeleton is to be elucidated by cloning the responsible terpene synthase gene in the future.

## 3. Materials and Methods

### 3.1. General Procedures

IR spectra were measured using an FT/IR-4100 spectrometer (JASCO, Tokyo, Japan). Ultraviolet (UV) spectra were obtained using a V-530 spectrometer (JASCO). CD spectrum was taken on a J-720WN spectrometer (JASCO). Specific rotations were recorded on a DIP-370 spectrometer (JASCO). High-resolution mass spectra (HRMS) were acquired on a Mariner Biospectrometry Workstation (Thermo Fisher Scientific (former Applied Biosystems), Waltham, MA, USA) in the positive electrospray ionization (ESI) mode using 80% MeOH-0.1% HCOOH and 80% MeOH-1 mM HCOONa for enhygromic (**1**) acid and deoxyenhygrolides (**2** and **3**), respectively, as the infusion solvent. NMR spectra were obtained on an Avance 400 (400 MHz) or Avance III HD 600 Cryo-Probe (600 MHz) spectrometer (Bruker BioSpin, Yokohama, Japan). The chemical shifts (ppm) were referenced to the solvent (DMSO-*d*_6_ or C_6_D_6_) peaks. Flash column chromatography was carried out with a medium-pressure gradient system equipped with a Pump Module C-605 and a Pump Manager C-615 (BÜCHI, Flawil, Switzerland). Preparative high-performance liquid chromatography (HPLC) was performed on a high-pressure gradient system composed of a PU-2087 pump, a DG-2080-53 degasser, an MX-2080-32 mixer, and a UV-2075 detector (JASCO). Melting point was measured on a micro melting point apparatus MP-J3 (Yanaco, Kyoto, Japan).

### 3.2. Bacterial Strain

The myxobacterial strain SNB-1 was isolated from a sand sample collected in September 1998 at a beach in Kashiwazaki, Niigata, Japan (37°22′ N, 138°33′ E). The isolation procedure was performed according to the method previously described [14]. The strain was cultured and maintained in N1.0-S75-15 agar or VY/2-S75-15 agar [17]. The growth of the strain was observed with 0.0–4.5% (*w*/*v*) NaCl (optimum, 1.0–2.0%). The 16S rRNA gene sequence (1505 nt) was deposited under the DDBJ accession number of LC066681. The phylogenetic tree (Figure 2) was constructed with the MEGA6 software by neighbor-joining method (NJ) based on 16S rRNA gene sequences, indicating that the strain SNB-1 belonged to the suborder *Nannocystineae*, order *Myxococcales*, in the class *Deltaproteobacteria* and was most closely related to the type strain of marine myxobacteria, *Enhygromyxa salina* SHK-1^T^ with a 97% similarity (Figure 2). The strain contained menaquinone-7 (MK-7) as the major respiratory quinone. Based on these findings, the strain was identified as a new species of the genus *Enhygromyxa*, and tentatively designated as *Enhygromyxa niigataensis.*

### 3.3. Isolation of ***1**–**3***

The marine myxobacterium *Enhygromyxa* sp. SNB-1 was precultured on the VY/2-SWS agar (5 g of Baker’s yeast cake, 20 g of sodium chloride, 0.5 mg of cyanocobalamin, and 15 g of agar (per liter of Sea Water Salt solution (SWS) [14])) supplemented with 0.1% (*w*/*v*) of sodium acetate for two weeks at 30 °C. The colony was cut into small pieces and inoculated into a 2 L Erlenmeyer flask containing 750 mL of VY/4-SWS liquid medium (2.5 g of Baker’s yeast cake, 20 g of sodium chloride, and 0.5 g of cyanocobalamin (per liter of SWS)) supplemented with 100 mM sodium acetate and gel cube for the bacterial growth promotion, then cultured for three weeks at 30 °C and 180 rpm. A total of 21 L of cultured broth containing the cells and gel cube was extracted once with 63 L and then twice with 10.5 L of acetone. The mixtures were filtered, combined, and then concentrated under reduced pressure. The residual aqueous mixture (a total of 14 L) was extracted with EtOAc (a total of 14 L). The concentration of the organic layer gave the crude extract (1.9 g). This extract was chromatographed on a HI-FLASH^TM^ silica gel column (size 2 L, 45 g, Yamazen Co., Osaka, Japan) with a linear gradient of 20–100% (40 min) EtOAc in hexane at 20 mL/min. The fractions (60.1 mg) eluted with 34–38% EtOAc in hexane were combined and subjected to reversed phase HPLC (Develosil ODS-HG-5 (20 i.d. × 250 mm, Nomura Chemical Ltd., Seto, Japan), 90–100% MeOH (20 min, linear gradient), 8 mL/min, detection at 220 nm). The fraction (9.3 mg) eluted at 22–25 min was further subjected to preparative HPLC (Develosil ODS-HG-5 (20 i.d. × 250 mm, Nomura Chemical Ltd.), MeOH–MeCN–H_2_O (42.5:42.5:15), 10 mL/min, detection at 220 nm) to obtain 6.9 mg of enhygromic acid (**1**, *t*_R_ = 41 min). The fraction eluted with 20–34% EtOAc in hexane in the flash column chromatography were combined and separated by flash column chromatograph (HI-FLASH^TM^ silica gel column (size M, 14 g), 0–25% EtOAc in hexane (50 min, linear gradient), 6 mL/min). The fractions (272 mg) eluted with 7.5–10% EtOAc in hexane were purified by HPLC (Develosil ODS-HG-5 (20 i.d. × 250 mm, 80% MeOH, 9 mL/min, detection at 315 nm) to afford deoxyenhygrolides A (**2**, 4.5 mg, *t*_R_ = 46 min) and B (**3**, 17.6 mg, *t*_R_ = 38 min).

#### 3.3.1. Enhygromic Acid (**1**)

Colorless powder; mp 209–213 °C (hexane), ${\left[\alpha \right]}_{D}^{26}$ −34 (*c* 0.06, MeOH); UV (1% MeOH in MeCN) λ_max_ (ε) 223 (11,000) nm; CD (1% MeOH in MeCN) λ_ext_ (Δε) 226 (−9.0), 196 (+8.0) nm; IR (film) ν_max_ 3640–2390 (br), 3084, 1685, 888, 757 cm^−1^; HR ESIMS *m/z* 303.2299 [M + H]^+^ (calcd. for C_20_H_31_O_2_ 303.2319).

#### 3.3.2. Deoxyenhygrolide A (**2**)

Pale yellow oil; UV (MeCN) λ_max_ (ε) 229 (14,000), 329 (32,000) nm; IR (film) ν_max_ 3060, 3028, 1763, 1027, 693 cm^−1^; HR ESIMS *m/z* 341.1511 [M + Na]^+^ (calcd. for C_22_H_22_O_2_Na 341.1512).

#### 3.3.3. Deoxyenhygrolide B (**3**)

Colorless oil; UV (MeCN) λ_max_ (ε) 299 (27,000) nm; IR (film) ν_max_ 3060, 3028, 1760, 1040, 723, 699 cm^−1^; HR ESIMS *m/z* 341.1499 [M + Na]^+^ (calcd. for C_22_H_22_O_2_Na 341.1512).

### 3.4. Conformational Analysis

The conformational analysis of enhygromic acid (**1**) was performed by Chem3D Ultra in ChemOffice 15.1 package (PerkinElmer Co., Waltham, MA, USA). The dihedral angle C-2–C-3–C-4–C-5 of **1** was rotated by every 15°, and MM2 energy minimization was carried out for all angles to obtain only two local minimum conformers. The result was visualized by a graph of the local minimum energy and angle against the input dihedral angle of C-2–C-3–C-4–C-5 (Figure S10).

### 3.5. Cytotoxicity Test

Cytotoxicity test against HeLa-S3 and B16 melanoma cells followed the methods reported previously [8,18].

### 3.6. NGF-Enhancing Activity for PC12 Cells

Nerve growth factor (NGF)-enhancing activity was evaluated by following the method reported previously [19] with some modifications. Briefly, PC12 cells cultured in DMEM supplemented with 10% fetal bovine serum (Biosera, Kansas City, MO, USA), 5% horse serum (Thermo Fisher Scientific Inc., Waltham, MA, USA), and antibiotics (100 μg/mL streptomycin and 100 units/mL penicillin). A total of 15,000 cultured cells in 1 mL of the same medium were seeded into each well of a 24-well microplate collagen Type I-coated (IWAKI, Tokyo, Japan). After preincubation for 24 h at 37 °C in an atmosphere of 5% CO_2_, the medium was replaced with 1 mL of the serum-free DMEM containing 0.5 ng/mL NGF (PeproTech, Rocky Hill, NJ, USA) and a compound in 5 μL DMSO. The morphological change of the cells was monitored by an inverted microscope FSX100 (OLYMPUS, Tokyo, Japan) after 4 days of incubation. A field that covers approximately 100 cells was randomly chosen and the number of the cells bearing neurite outgrowth longer than the cell diameter was counted. The data were the mean ± SD of triplicate.

### 3.7. Anti-Phytophthora Assay

The growth inhibitory activity against the oomycete *Phytophthora capsici* was evaluated by following the method reported previously [8].

### 3.8. MIC Assay

All microorganisms, *E. coli* AJ 3837 (NBRC 14237), *B. subtilis* AJ 12865 (ATCC 6051), *C. rugosa* AJ 14513 (NBRC 0750), *A. niger* AJ 117065 (ATCC 10864), *R. oryzae* AJ 117321 (JCM 5582), and *T. mentagrophytes* AJ 117161 (NBRC 7522), were provided by the Institute for Innovation, Ajinomoto Co., Inc. (Kanagawa, Japan). Antibacterial tests against *E. coli* and *B. subtilis* followed the reported protocol [20]. Antifungal activity was evaluated by following the reported methods [21,22,23]. The tests were carried out at the concentrations of 1, 2, 4, 8, 16, 32, and 64 μg/mL for bacteria or 0.5, 1, 2, 4, 8, 16, and 32 μg/mL for fungi.

## 4. Conclusions

Three new compounds, enhygromic acid (**1**) and deoxyenhygrolides (**2** and **3**), were isolated from a new marine myxobacterial species of the genus *Enhygromyxa*, and the absolute configuration of **1** was determined by NOESY and CD spectrum. As regards the results of the biological tests, **1** showed the cytotoxicity against B16 melanoma cells, NGF neurite outgrowth promoting activity to PC12 cells, and growth inhibition against *B. subtilis*. On the other hand, **2** and **3** did not show any biological activities in this study. Compound **1** has a quite unique and rare acrylic acid derivative with a decahydroacenaphthylene skeleton, which had not been reported prior to this study. Therefore, **1** will be noticed in the field of organic synthesis or biosynthesis research in the future.

## Figures and Tables

**Table 1:** ^1^H (600 MHz) and ^13^C (150 MHz) NMR spectroscopic data for enhygromic acid (**1**) in DMSO-*d*_6_.

Position	δ_C_, Type	δ_H_ (*J* in Hz)	HMBC *^a^*
1	170.2, C		
2	127.2 (br), C		
3	145.9 (br), CH	7.27, s	4
4	52.2, C		
5	46.7, CH	2.81, brd (7.1)	4
6	148.9, C		
7a	35.3, CH_2_	2.08, m	
7b		2.39, dt (13.1, 3.0)	
8a	21.7, CH_2_	1.36, m	
8b		1.55 m	
9	45.2, CH	2.04, m	4
10	31.7, CH	1.55, m	
11a	29.4, CH_2_	1.09, m	
11b		1.42, m	
12	21.6, CH_2_	1.43, m; 1.45, m	4
13	46.9, CH	1.49, dd (12.7, 3.4)	
14	35.6, C		
15a	40.6, CH_2_	1.35, m	4
15b		1.92, m	
16a	108.1, CH_2_	4.76, s	5, 6, 7
16b		4.85, d (1.5)	
17	19.6, CH_3_	0.76, d (6.9)	
18	32.6, CH_3_	1.00, s	13, 14
19	26.7, CH_3_	0.86, s	14, 15
20	14.7, CH_3_	1.92, s	1, 2, 3

*^a^* HMBC correlations are from proton(s) started to the indicated carbon.

**Table 2:** ^1^H (400 MHz) and ^13^C (100 MHz) NMR spectroscopic data for deoxyenhygrolides A (**2**) and B (**3**) in C_6_D_6_.

	Deoxyenhygrolide A (2)			Deoxyenhygrolode B (3)		
Position	δ_C_, Type	δ_H_ (*J* in Hz)	HMBC *^a^*	δ_C_, Type	δ_H_ (*J* in Hz)	HMBC *^a^*
1	169.8, C			169.3, C		
2	127.5, C			132.5, C		
3	152.0, C			149.6, C		
4	149.2, C			150.2, C		
5	109.0, CH	5.70, s	3, 4, 6, 7, 11	113.8, CH	6.58, s	3, 4, 6, 7, 11
6	133.8, C			133.7, C		
7, 11	130.7, CH	7.74, d (7.5)		129.5, CH	6.82, m	
8, 10	129.0, CH	7.12, t (7.5)		128.3, CH	6.96, m	
9	128.7, CH	7.01, t (7.5)		128.0, CH	6.96, m	
12	30.1, CH_2_	3.51, s	1, 2, 3, 13, 14, 18	30.2, CH_2_	3.56, s	1, 2, 3, 13, 14, 18
13	138.4, C			138.4, C		
14, 18	128.9, CH	7.18, d (7.5)		128.9, CH	7.25, d (7.5)	
15, 17	128.9, CH	7.10, t (7.5)		128.9, CH	7.12, t (7.2)	
16	126.8, CH	7.02, t (7.5)		126.9, CH	7.02, t (7.4)	
19	33.6, CH_2_	1.92, d (7.4)	2, 3, 4	34.8, CH_2_	2.00, d (7.1)	2, 3, 4
20	29.1, CH	1.55, non (6.6)	3	28.3, CH	1.07, non (6.8)	3
21, 22	22.6, CH_3_	0.62, d (6.6)		22.0, CH_3_	0.33, d (6.6)	

*^a^* HMBC correlations are from proton(s) started to the indicated carbon.

## Supplementary Materials

The following are available online at www.mdpi.com/1660-3397/15/4/109/s1: Figure S1: ^1^H NMR spectrum of **1** (600 MHz, DMSO-*d*_6_), Figure S2: ^13^C NMR spectrum of **1** (150 MHz, DMSO-*d*_6_), Figure S3: DQF-COSY of **1** (600 MHz, DMSO-*d*_6_), Figure S4: HSQC spectrum of **1** (600 MHz, DMSO-*d*_6_), Figure S5: HMBC spectrum of **1** (600 MHz, DMSO-*d*_6_), Figure S6: NOESY of **1** (600 MHz, DMSO-*d*_6_), Figure S7: UV spectrum of **1**, Figure S8: IR spectrum of **1**, Figure S9: ESI-TOF-MS (+) spectrum of **1**, Figure S10: Local minimum energy (orange line) and dihedral angle C2–C3–C4–C5 (blue dotted line) against the input dihedral angle in **1**, Figure S11: ^1^H NMR spectrum of **2** (400 MHz, C_6_D_6_), Figure S12: ^13^C NMR spectrum of **2** (100 MHz, C_6_D_6_), Figure S13: DQF-COSY of **2** (400 MHz, C_6_D_6_), Figure S14: HSQC spectrum of **2** (400 MHz, C_6_D_6_), Figure S15: HMBC spectrum of **2** (400 MHz, C_6_D_6_), Figure S16: NOESY of **2** (400 MHz, C_6_D_6_), Figure S17: UV spectrum of **2**, Figure S18: IR spectrum of **2**, Figure S19: ESI-TOF-MS spectrum of **2**, Figure S20: ^1^H NMR spectrum of **3** (400 MHz, C_6_D_6_), Figure S21: ^13^C NMR spectrum of **3** (100 MHz, C_6_D_6_), Figure S22: DQF-COSY of **3** (400 MHz, C_6_D_6_), Figure S23: HSQC spectrum of **3** (400 MHz, C_6_D_6_), Figure S24: HMBC spectrum of **3** (400 MHz, C_6_D_6_), Figure S25: NOESY of **3** (400 MHz, C_6_D_6_), Figure S26: UV spectrum of **3**, Figure S27: IR spectrum of **3**, Figure S28: ESI-TOF-MS spectrum of **3**.
