# Supplementary material for: An Unusual Diterpene—Enhygromic Acid and Deoxyenhygrolides from a Marine Myxobacterium, Enhygromyxa sp
Source: Mar Drugs. 2017 Apr 6;15(4):109. doi: 10.3390/md15040109 (PMC5408255; doi:10.3390/md15040109)
Supplement: Supplementary file 1 [file marinedrugs-15-00109-s001.pdf]

Supplementary Materials: An Unusual Diterpene−Enhygromic Acid and Deoxyenhygrolides from a Marine Myxobacterium, *Enhygromyxa* sp.

Tomohiko Tomura, Shiori Nagashima, Satoshi Yamazaki, Takashi Iizuka, Ryosuke Fudou and Makoto Ojika

Contents

*Spectral data and conformational analysis of enhygromic acid (**1**)*

Figure S1: ^{1}H NMR spectrum of **1** (600 MHz, DMSO-*d*$_6$), Figure S2: ^{13}C NMR spectrum of **1** (150 MHz, DMSO-*d*$_6$), Figure S3: DQF-COSY of **1** (600 MHz, DMSO-*d*$_6$), Figure S4: HSQC spectrum of **1** (600 MHz, DMSO-*d*$_6$), Figure S5: HMBC spectrum of **1** (600 MHz, DMSO-*d*$_6$), Figure S6: NOESY of **1** (600 MHz, DMSO-*d*$_6$), Figure S7: UV spectrum of **1**, Figure S8: IR spectrum of **1**, Figure S9: ESI-TOF-MS (+) spectrum of **1**, Figure S10: Local minimum energy (orange line) and dihedral angle C2–C3–C4–C5 (blue dotted line) against the input dihedral angle in **1**

*Spectral data of deoxyenhygrolide A (**2**)*

Figure S11: ^{1}H NMR spectrum of **2** (400 MHz, C_6D_6), Figure S12: ^{13}C NMR spectrum of **2** (100 MHz, C_6D_6), Figure S13: DQF-COSY of **2** (400 MHz, C_6D_6), Figure S14: HSQC spectrum of **2** (400 MHz, C_6D_6), Figure S15: HMBC spectrum of **2** (400 MHz, C_6D_6), Figure S16: NOESY of **2** (400 MHz, C_6D_6), Figure S17: UV spectrum of **2**, Figure S18: IR spectrum of **2**, Figure S19: ESI-TOF-MS spectrum of **2**

*Spectral data of deoxyenhygrolide B (**3**)*

Figure S20: ^{1}H NMR spectrum of **3** (400 MHz, C_6D_6), Figure S21: ^{13}C NMR spectrum of **3** (100 MHz, C_6D_6), Figure S22: DQF-COSY of **3** (400 MHz, C_6D_6), Figure S23: HSQC spectrum of **3** (400 MHz, C_6D_6), Figure S24: HMBC spectrum of **3** (400 MHz, C_6D_6), Figure S25: NOESY of **3** (400 MHz, C_6D_6), Figure S26: UV spectrum of **3**, Figure S27: IR spectrum of **3**, Figure S28: ESI-TOF-MS spectrum of **3**.

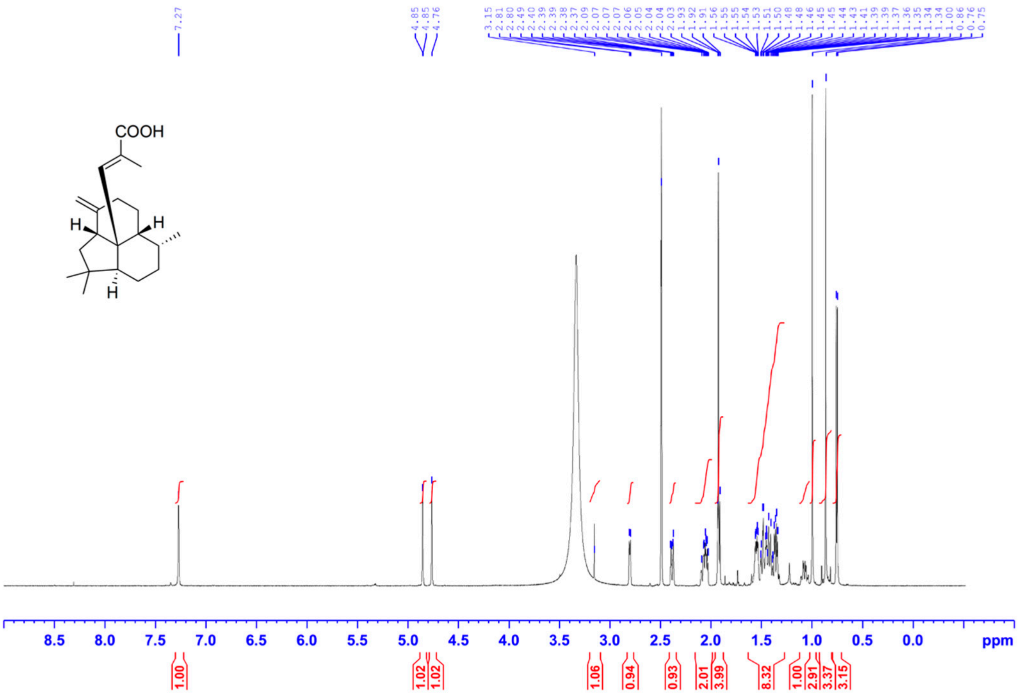

Figure S1. ^{1}H NMR spectrum of **1** (600 MHz, DMSO-*d*$_6$).

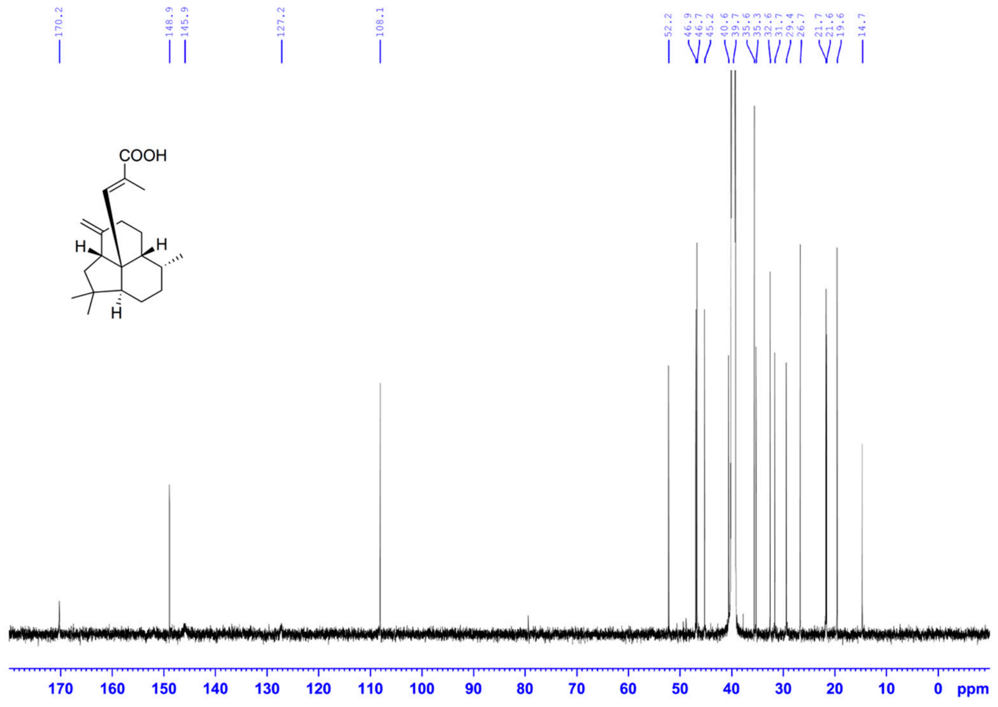

Figure S2. ^{13}C NMR spectrum of **1** (150 MHz, DMSO-d_6).

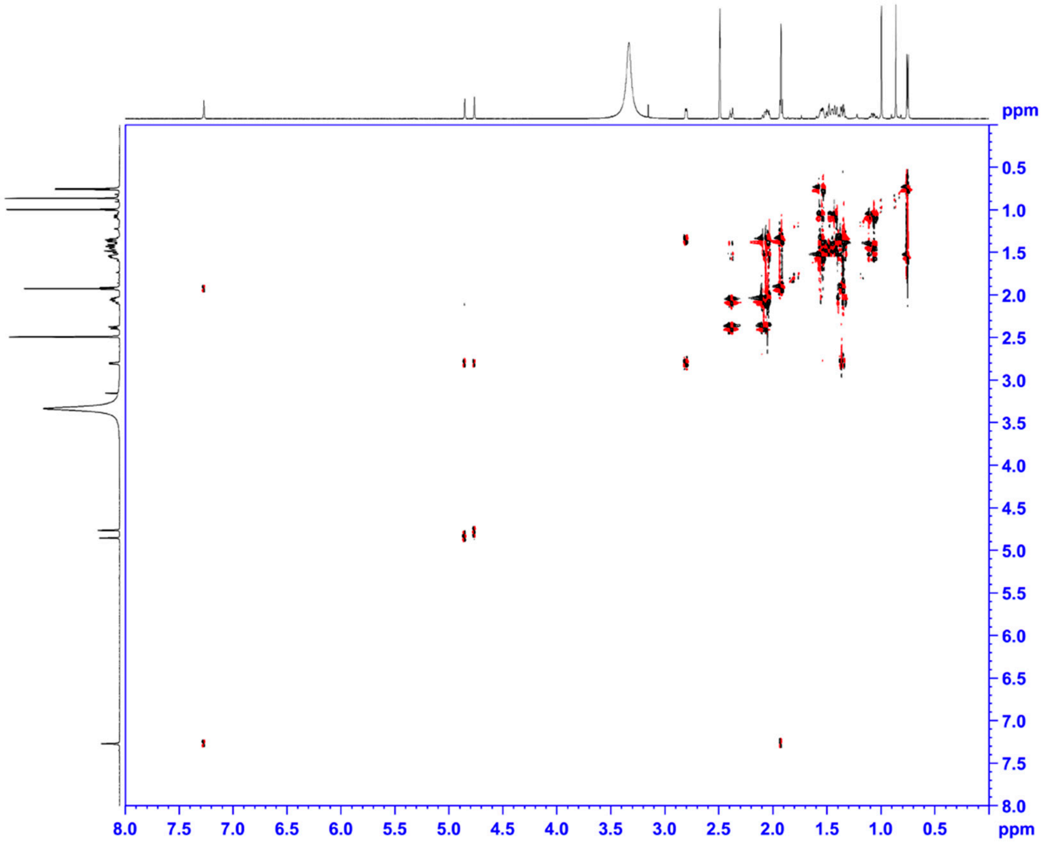

Figure S3. DQF-COSY of **1** (600 MHz, DMSO-d_6).

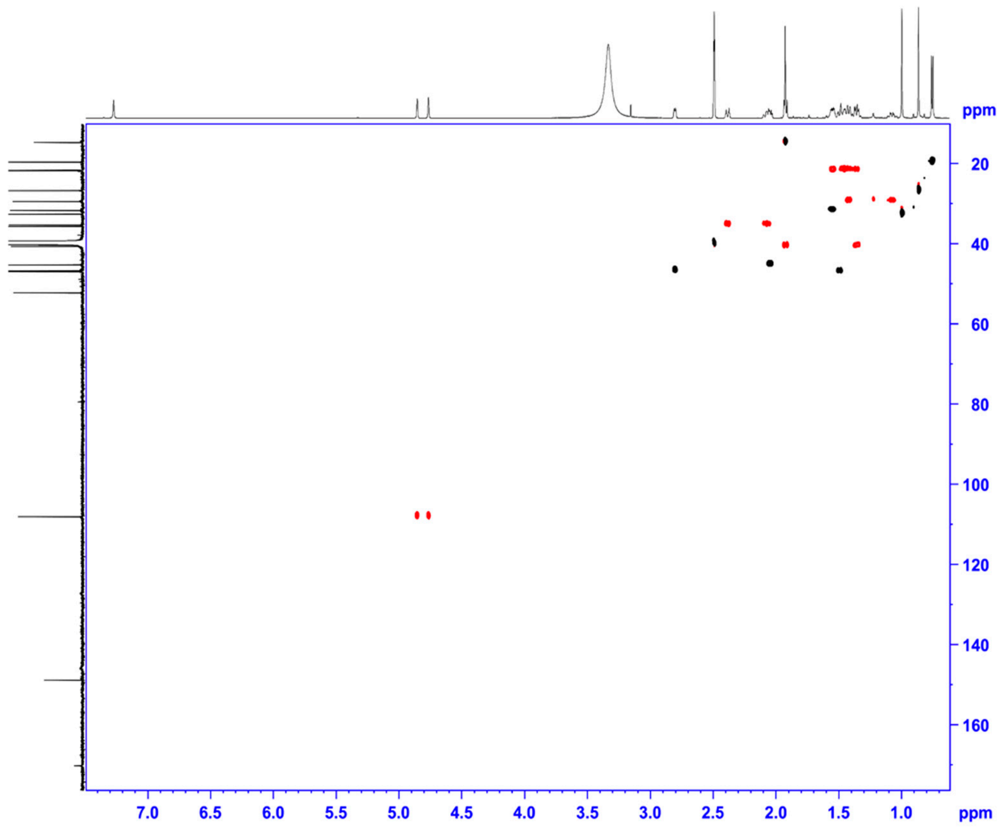

Figure S4. HSQC spectrum of **1** (600 MHz, DMSO-*d*6).

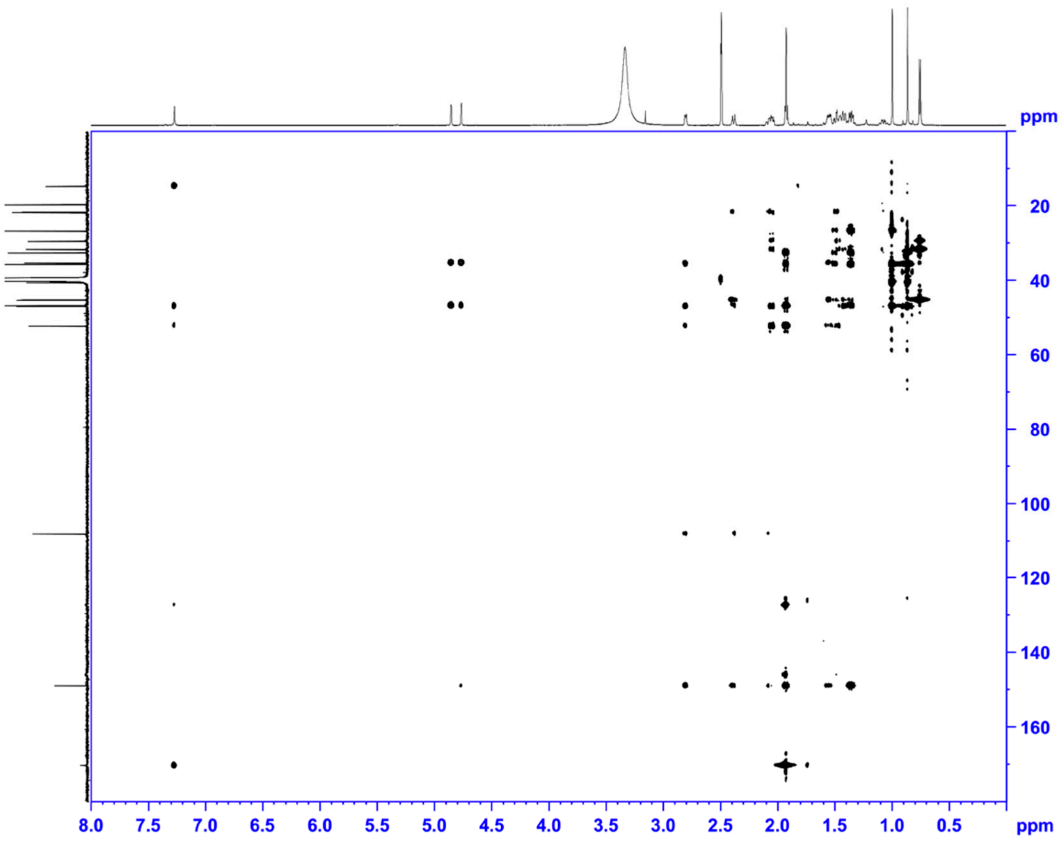

Figure S5. HMBC spectrum of **1** (600 MHz, DMSO-*d*6).

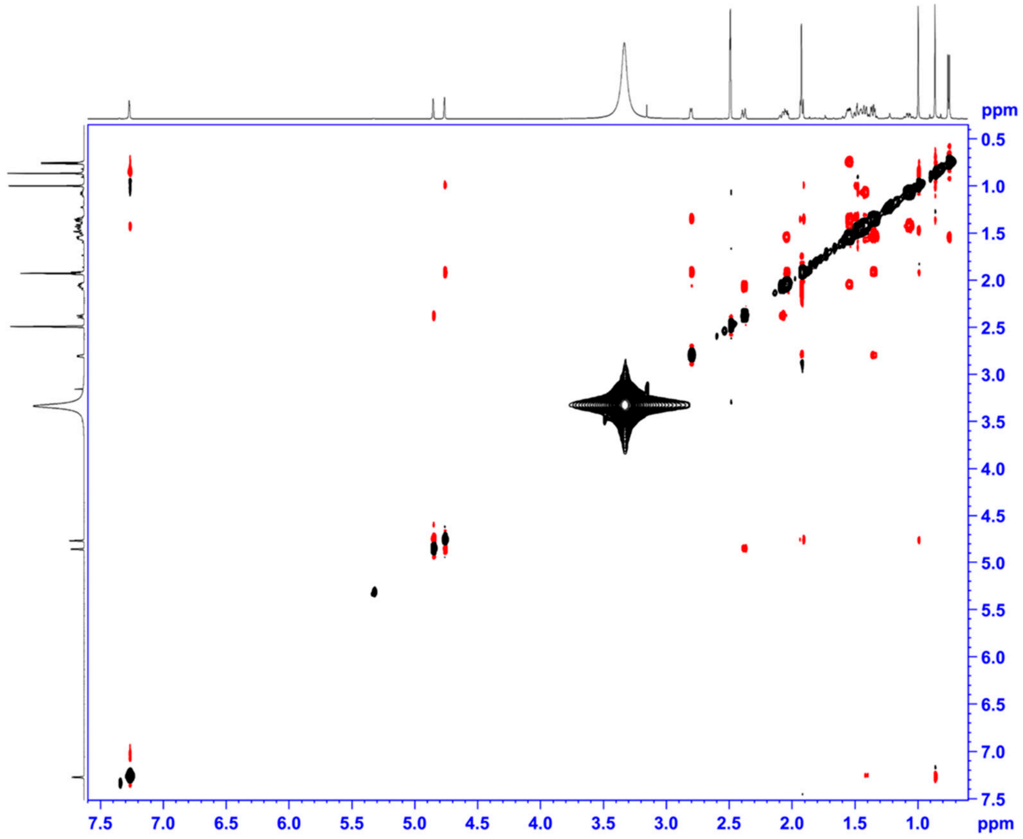

Figure S6. NOESY of **1** (600 MHz, DMSO-d_6).

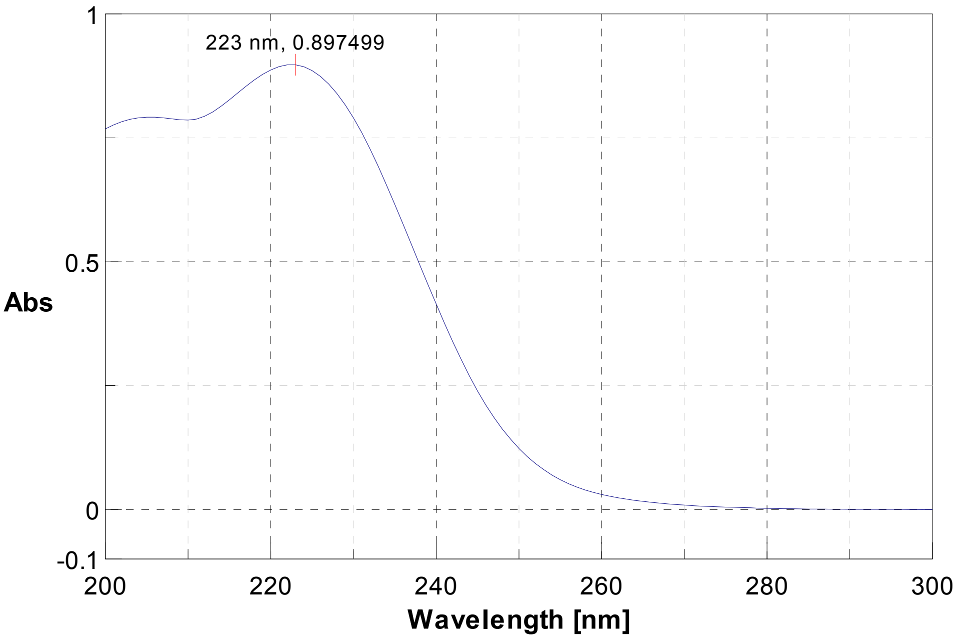

Figure S7. UV spectrum of **1**.

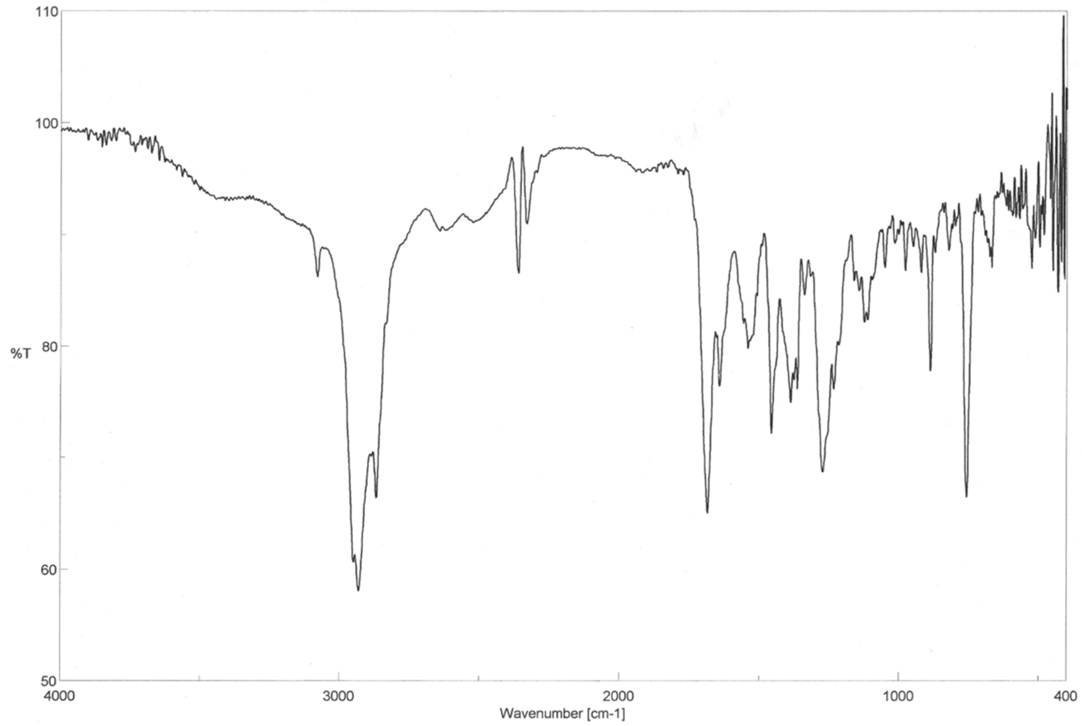

Figure S8. IR spectrum of **1**.

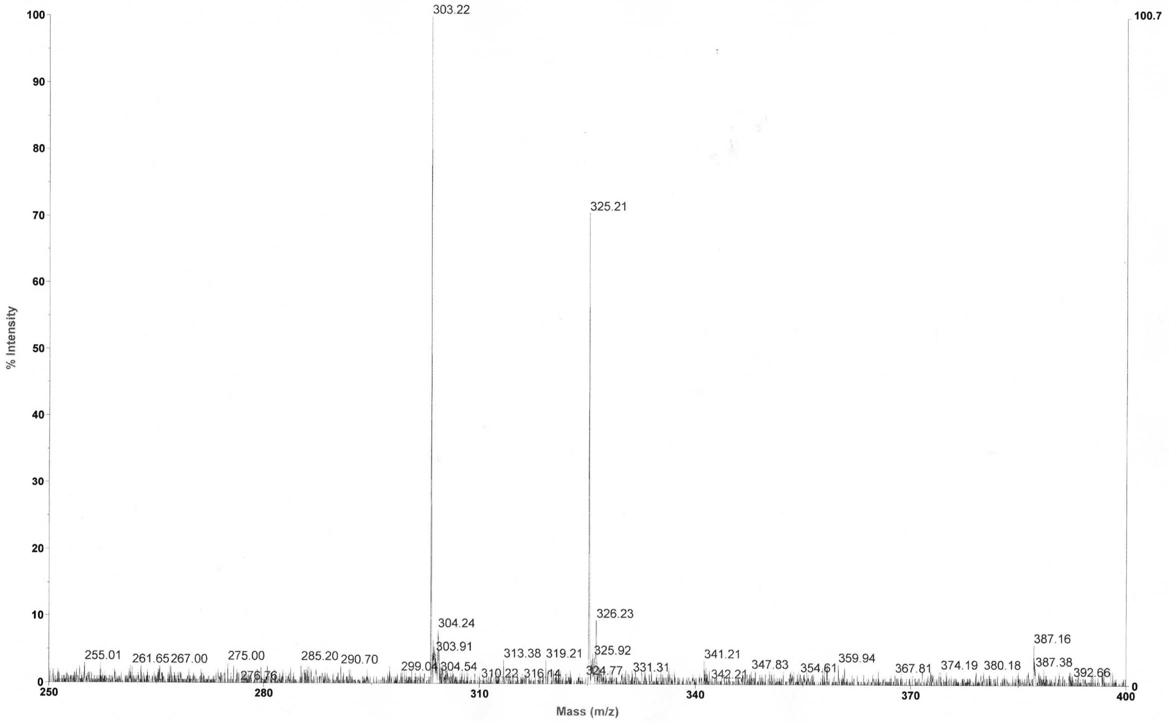

Figure S9. ESI-TOF-MS (+) spectrum of **1**.

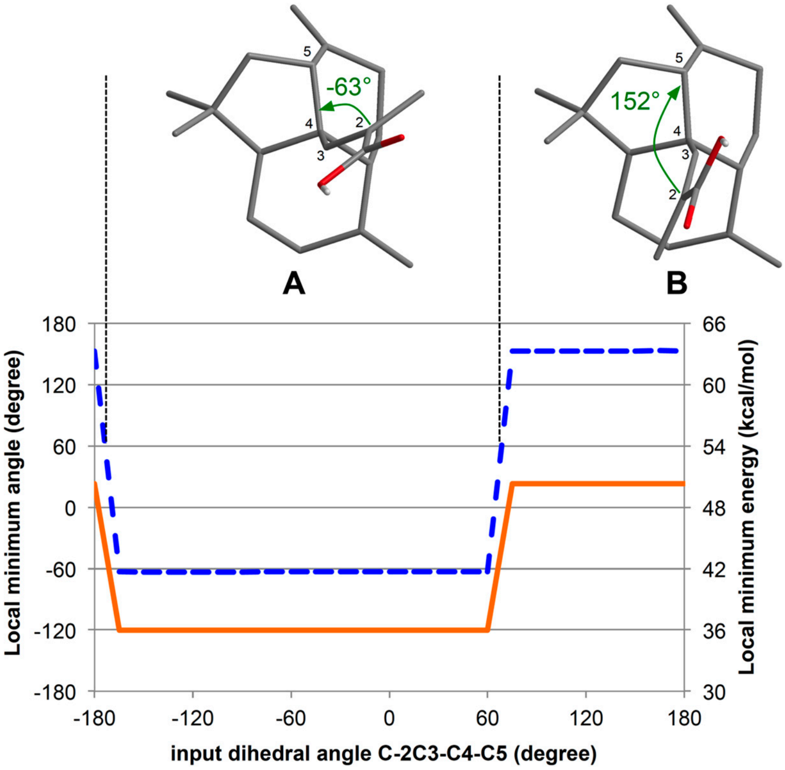

Figure S10. Local minimum energy (orange line) and dihedral angle C2–C3–C4–C5 (blue dotted line) against the input dihedral angle in **1**. Only two energy minimized conformers **A** and **B** were obtained.

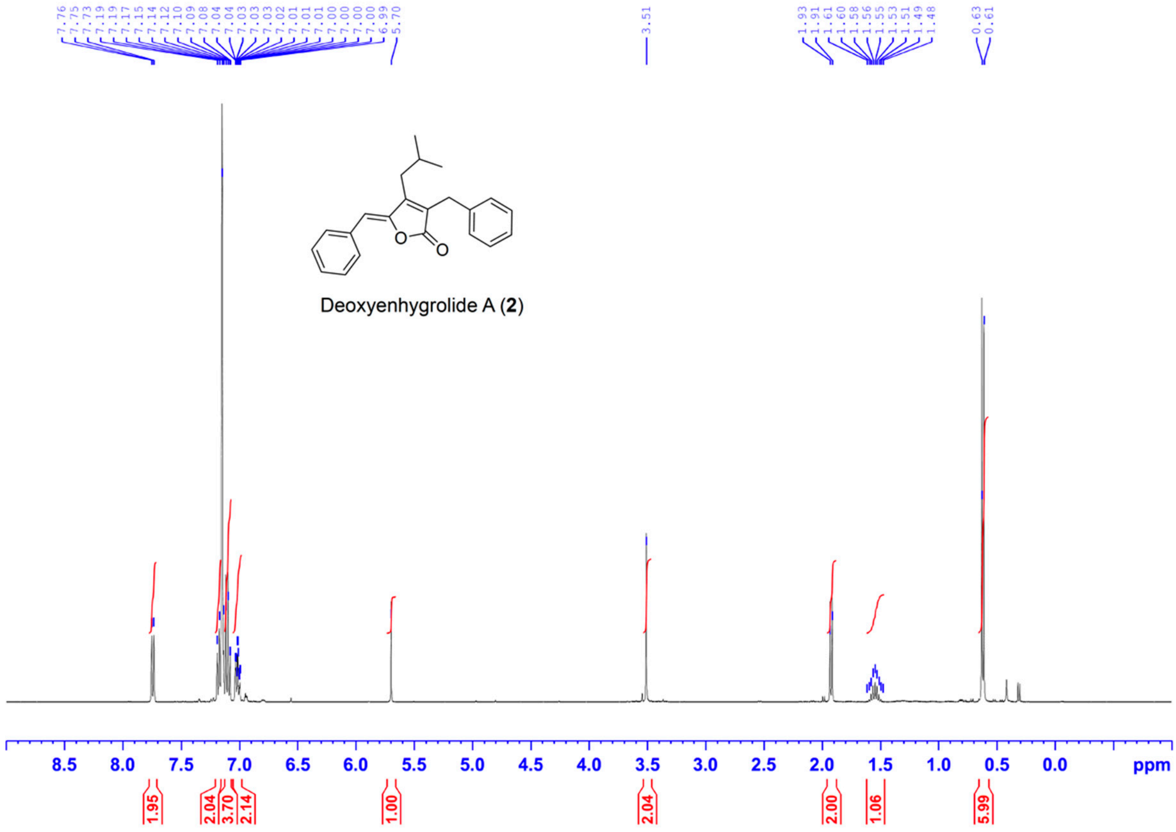

Figure S11. ^{1}H NMR spectrum of **2** (400 MHz, C_6D_6).

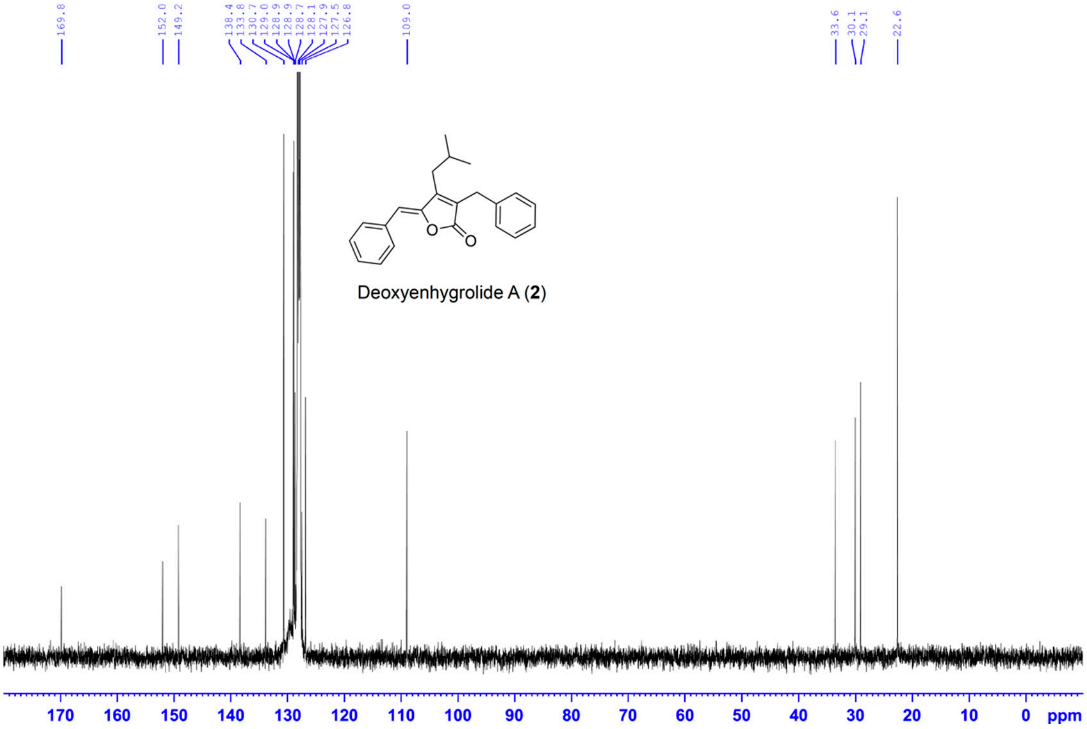

Figure S12. ^{13}C NMR spectrum of **2** (100 MHz, C_6D_6).

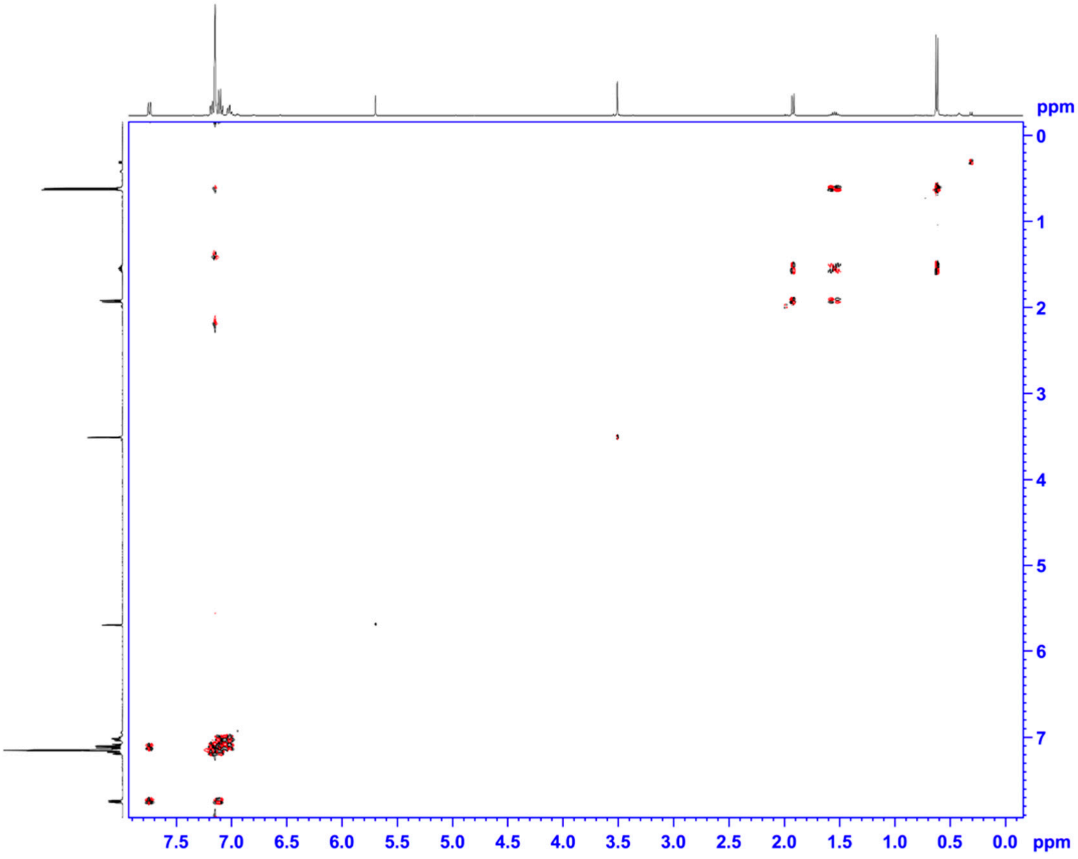

Figure S13. DQF-COSY of **2** (400 MHz, C_6D_6).

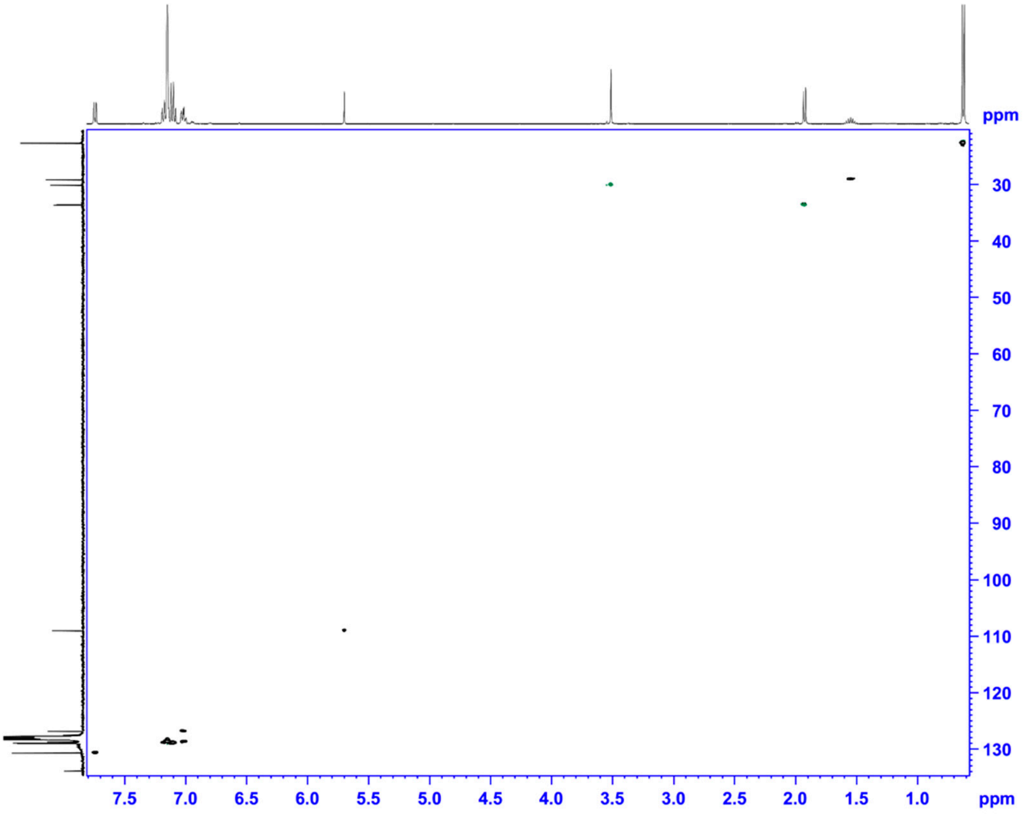

Figure S14. HSQC spectrum of **2** (400 MHz, C_6D_6).

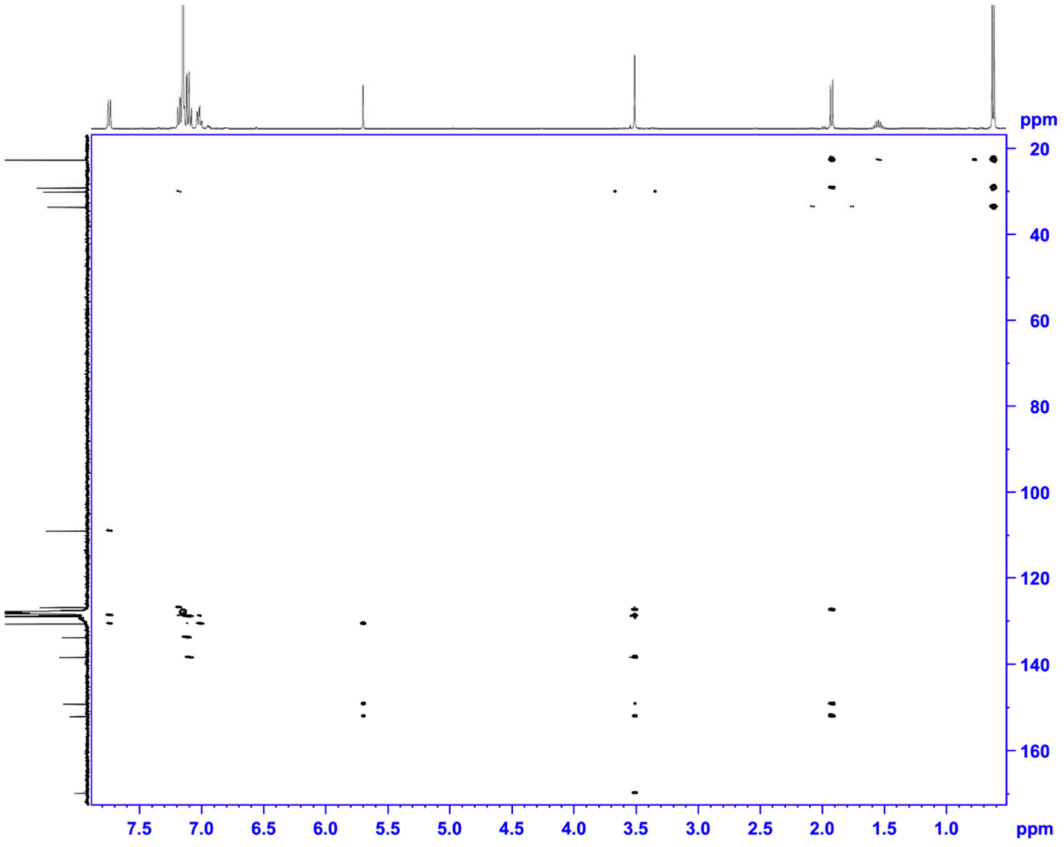

Figure S15. HMBC spectrum of **2** (400 MHz, C_6D_6).

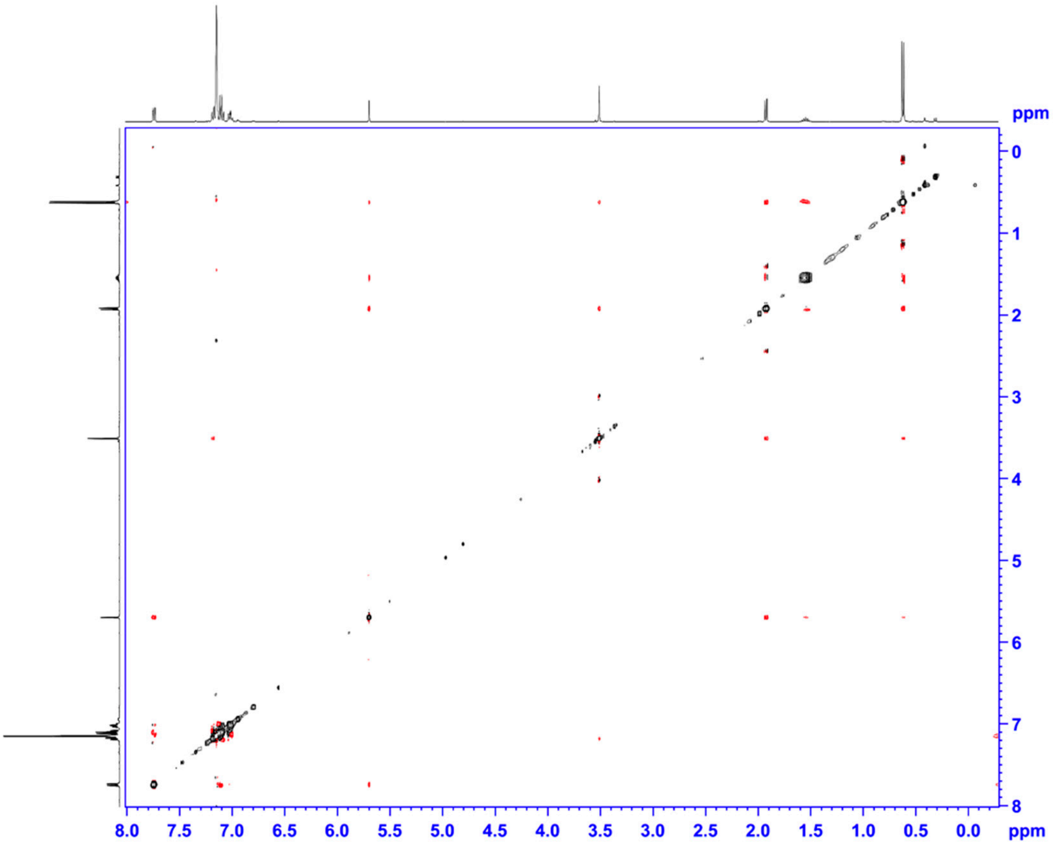

Figure S16. NOESY of **2** (400 MHz, C_6D_6).

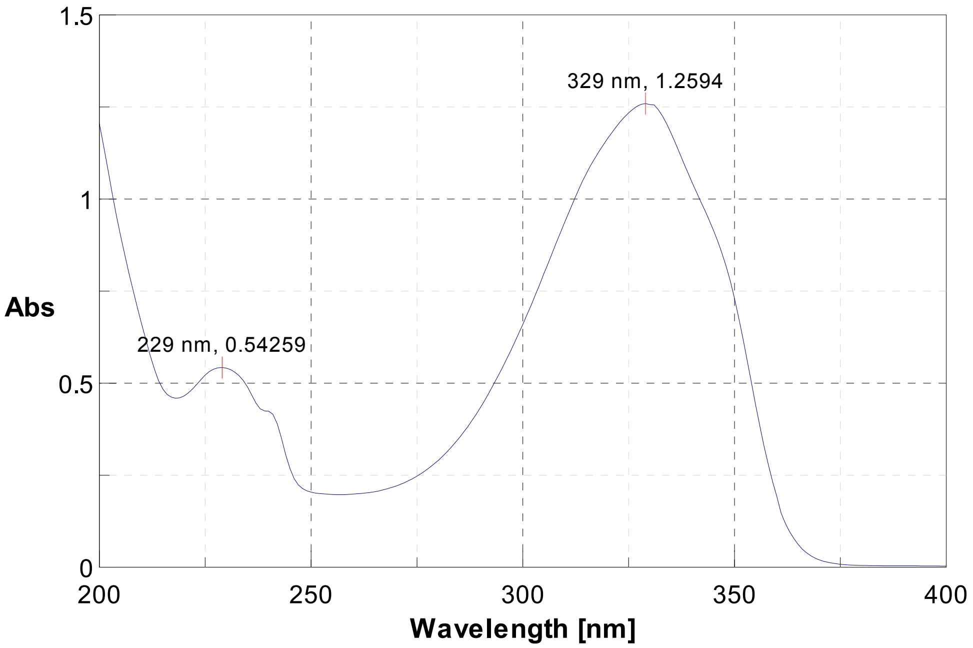

Figure S17. UV spectrum of **2**.

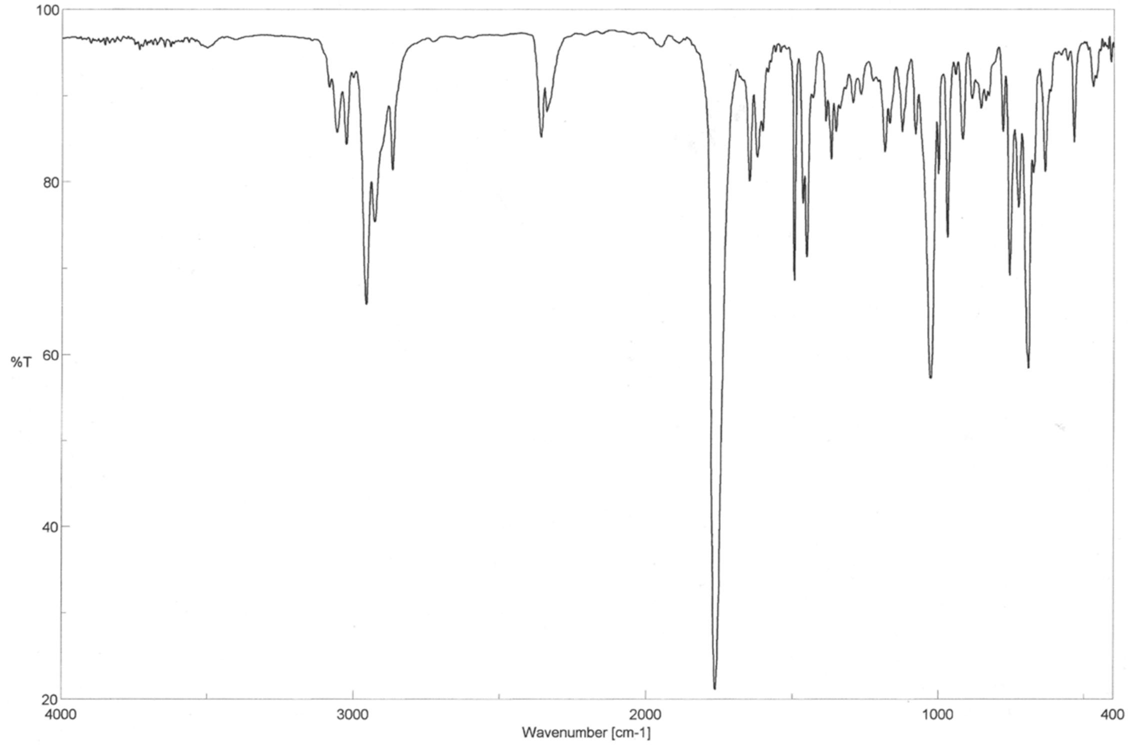

Figure S18. IR spectrum of **2**.

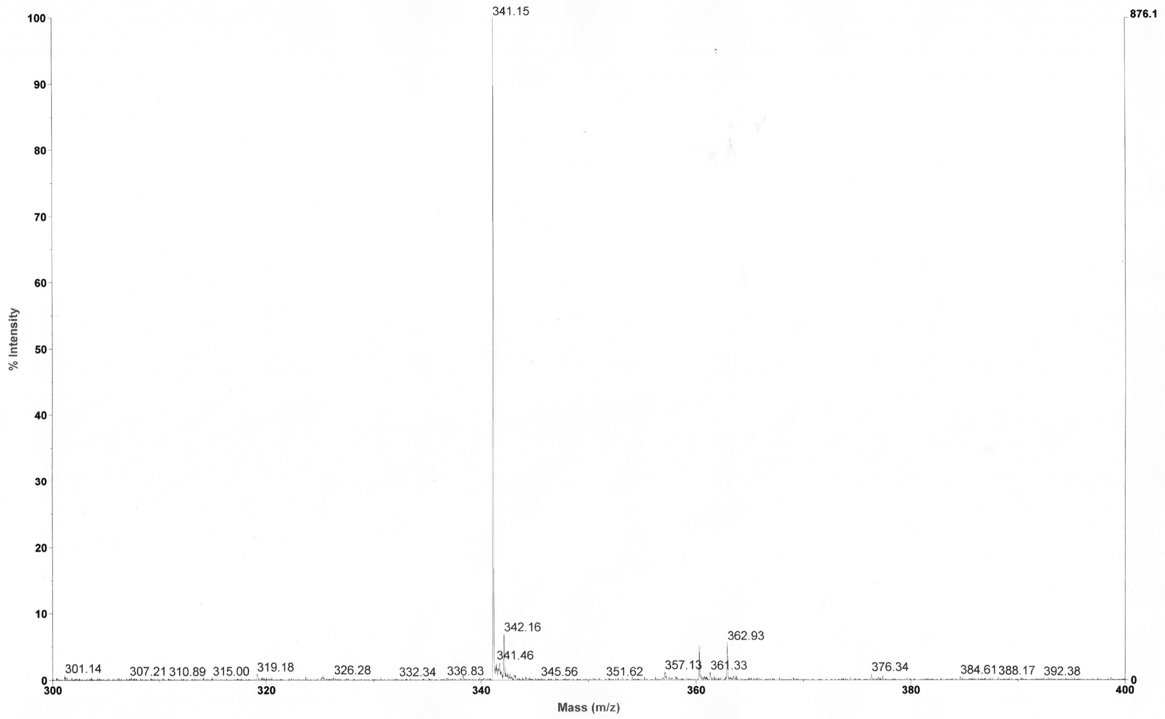

Figure S19. ESI-TOF-MS spectrum of **2**.

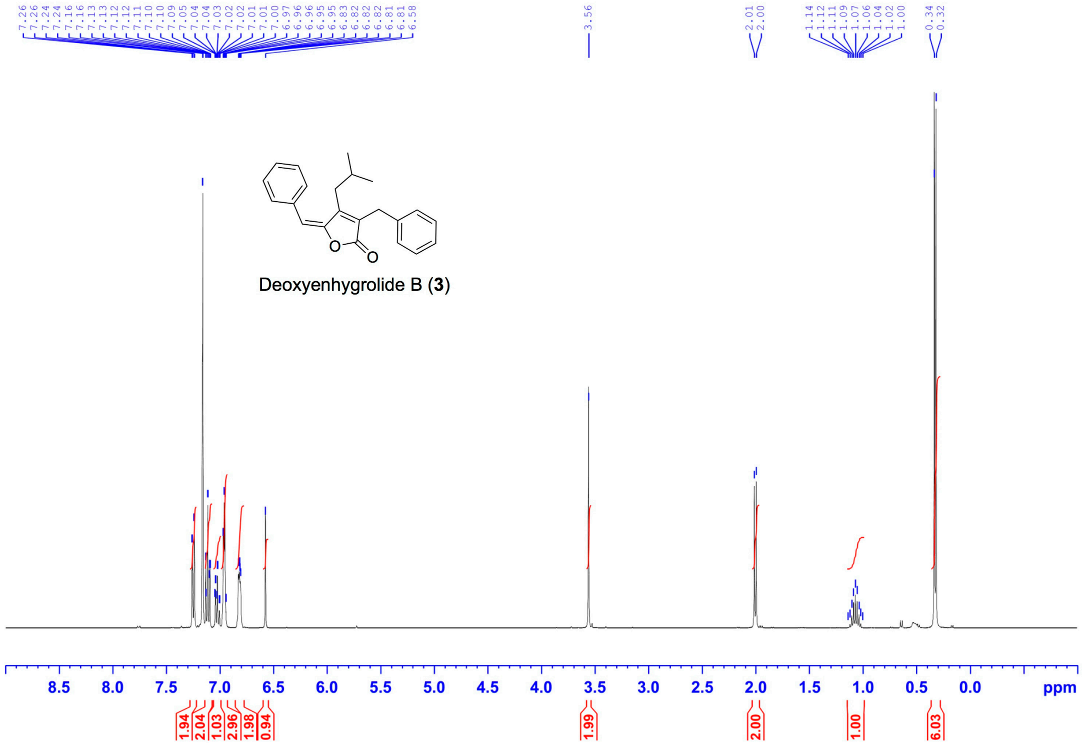

Figure S20. ^{1}H NMR spectrum of **3** (400 MHz, C_6D_6).

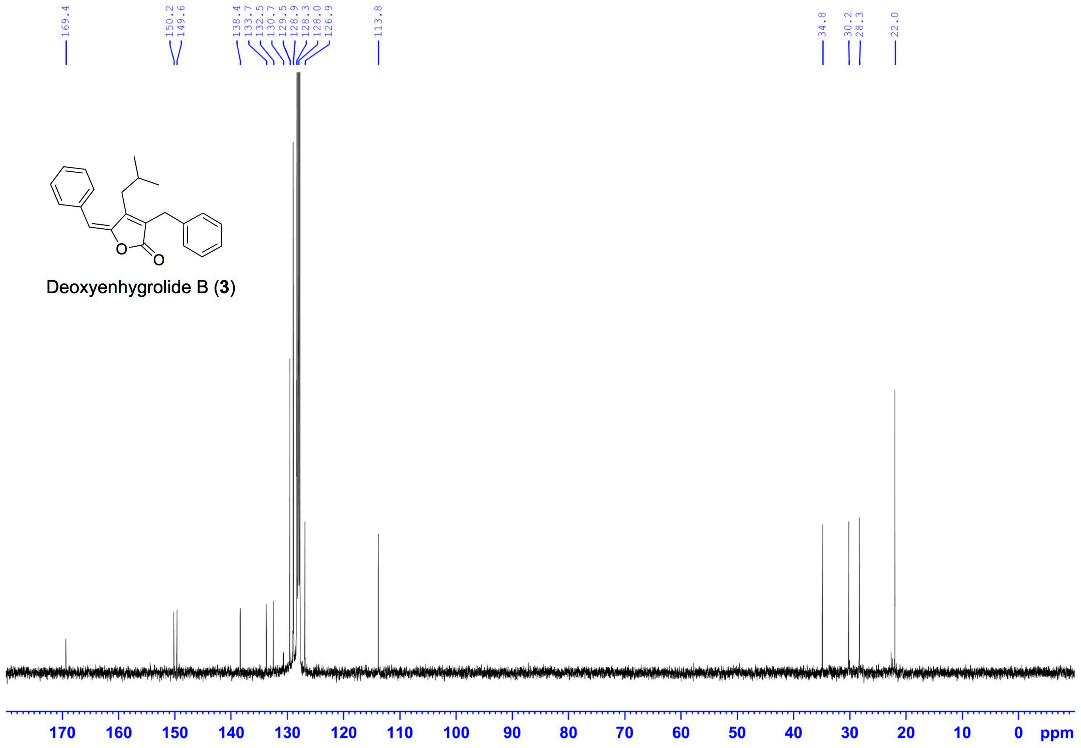

Figure S21. ^{13}C NMR spectrum of **3** (100 MHz, C_6D_6).

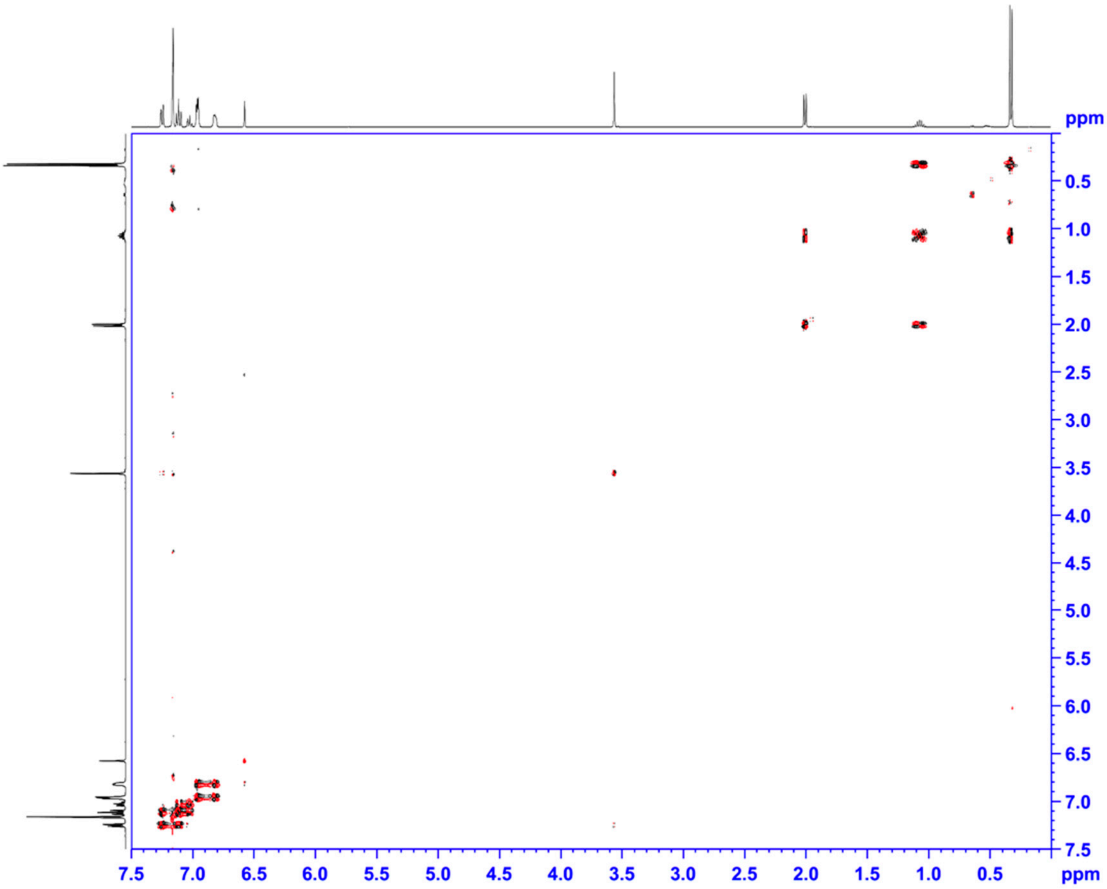

Figure S22. DQF-COSY of **3** (400 MHz, C_6D_6).

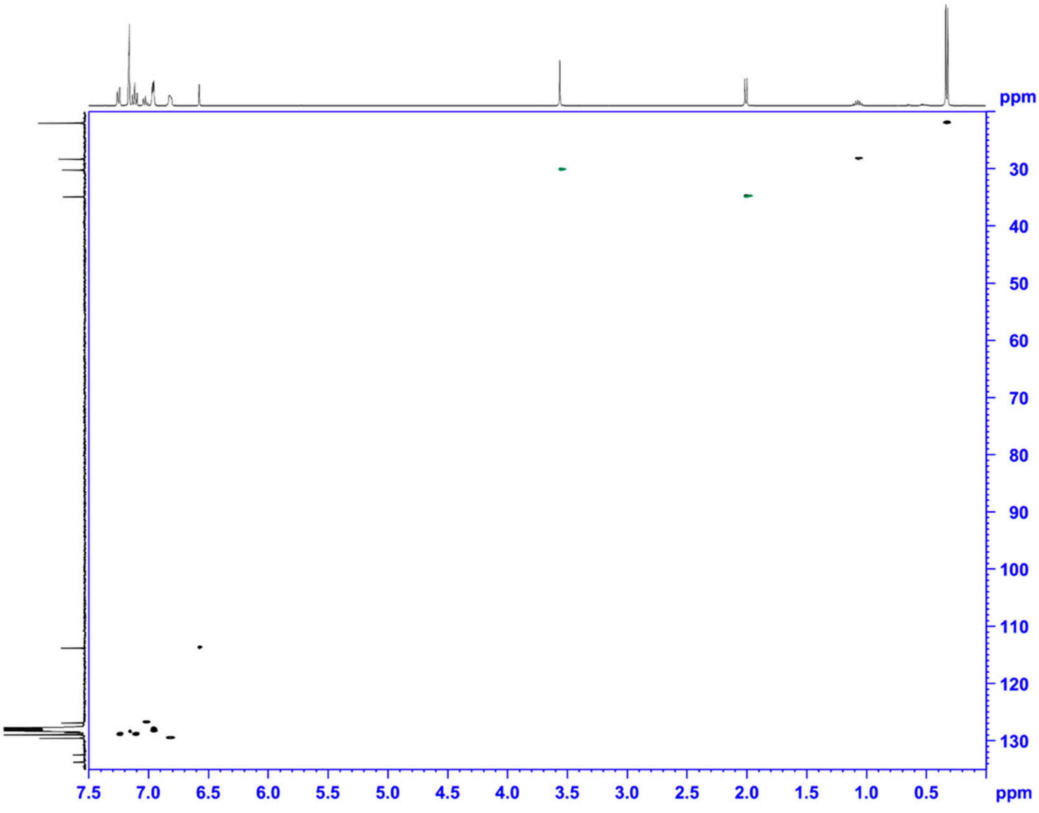

Figure S23. HSQC spectrum of **3** (400 MHz, C_6D_6).

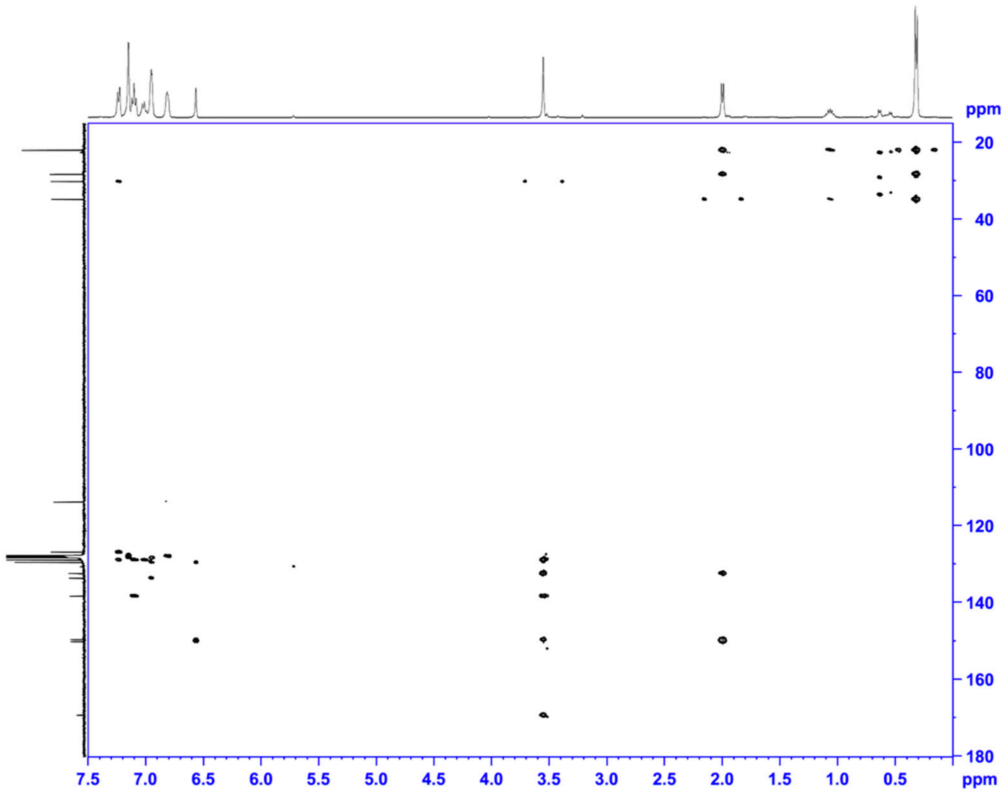

Figure S24. HMBC spectrum of **3** (400 MHz, C_6D_6).

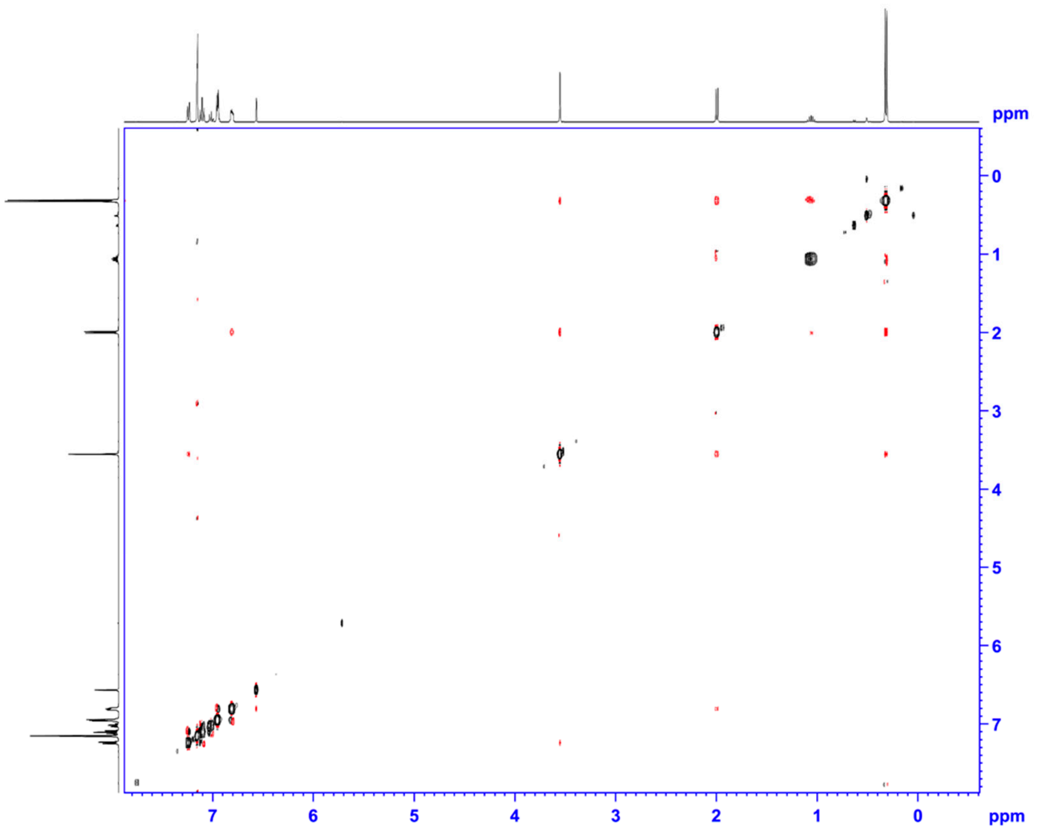

Figure S25. NOESY of **3** (400 MHz, C_6D_6).

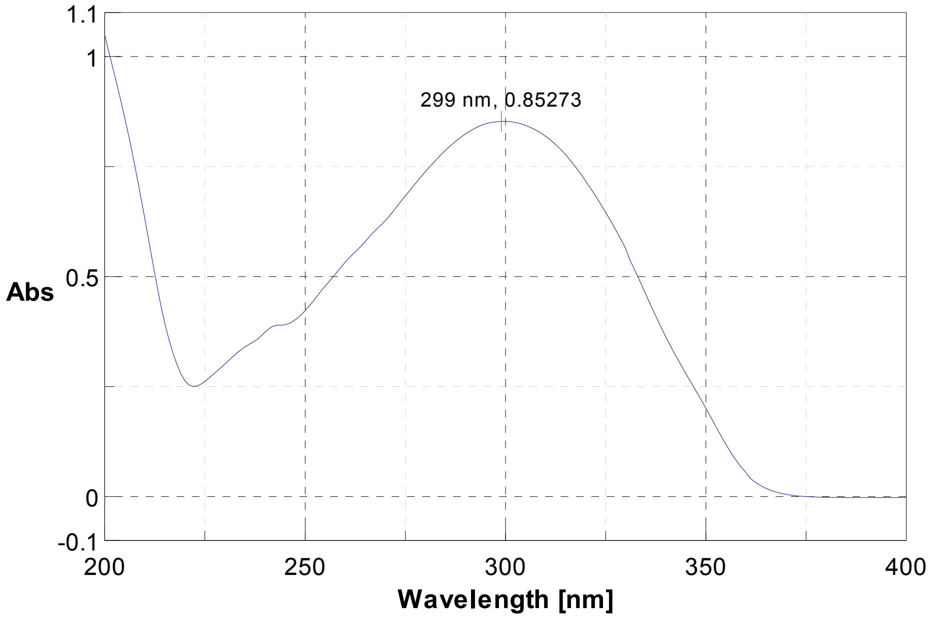

Figure S26. UV spectrum of **3**.

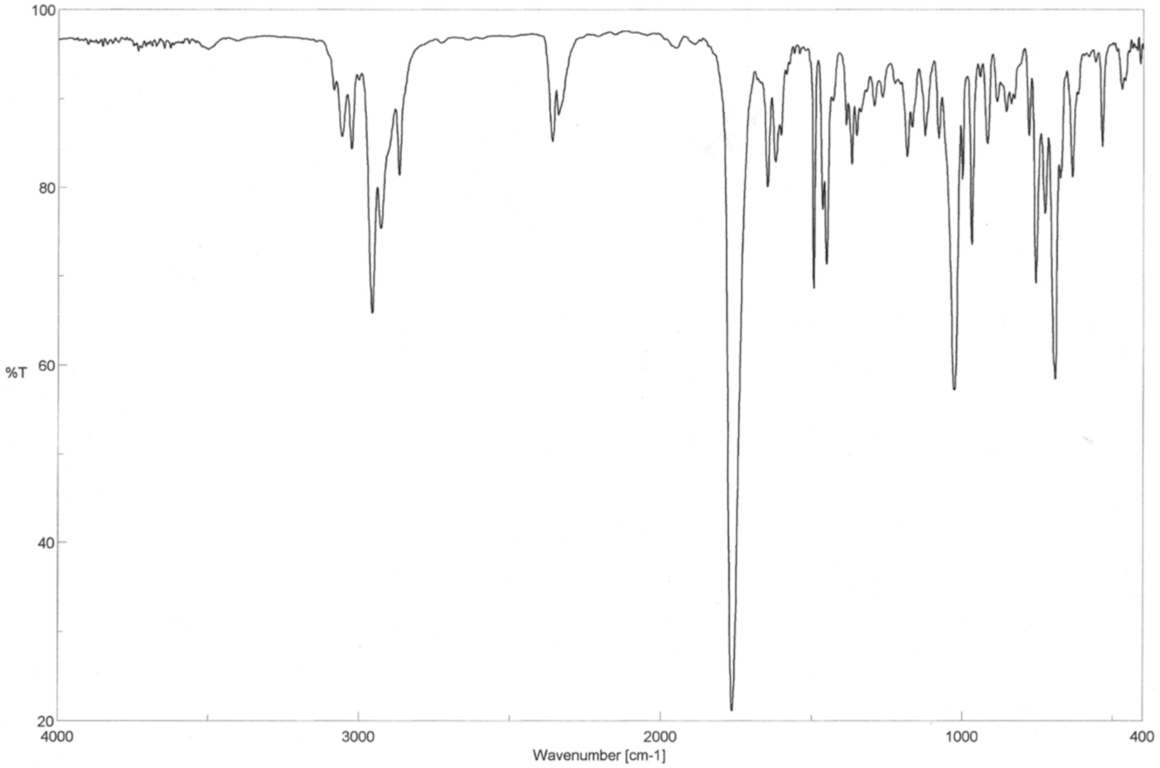

Figure S27. IR spectrum of **3**.

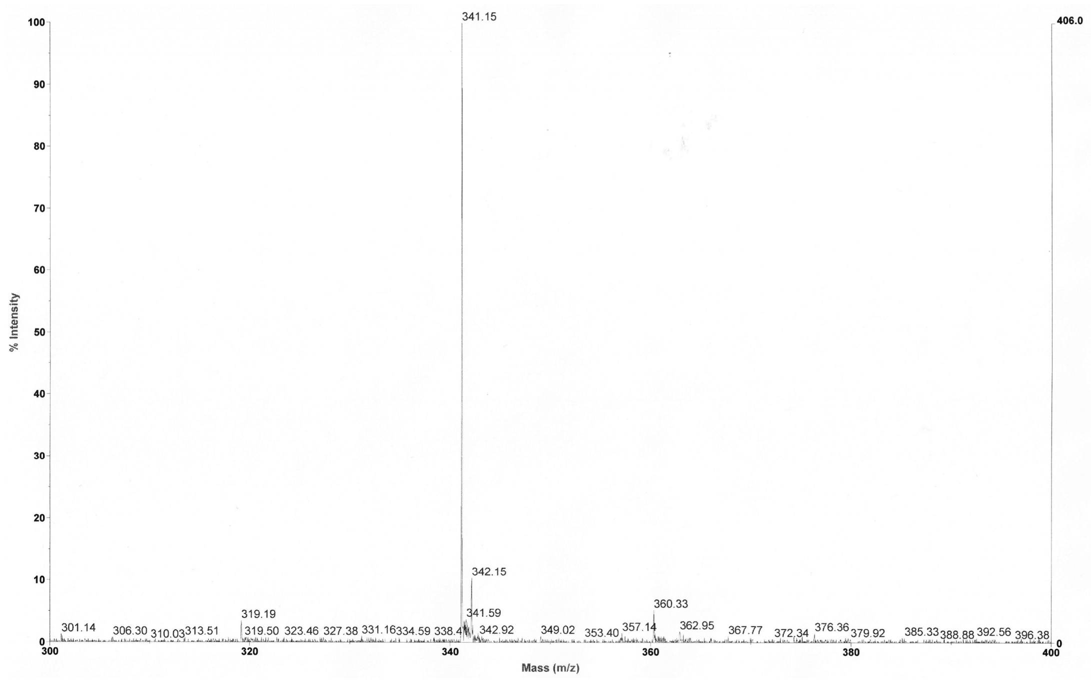

Figure S28. ESI-TOF-MS spectrum of **3**.
